# The alternatively-included 11a sequence modifies the effects of Mena on actin cytoskeletal organization and cell behavior

**DOI:** 10.1038/srep35298

**Published:** 2016-10-17

**Authors:** Michele Balsamo, Chandrani Mondal, Guillaume Carmona, Leslie M. McClain, Daisy N. Riquelme, Jenny Tadros, Duan Ma, Eliza Vasile, John S. Condeelis, Douglas A. Lauffenburger, Frank B. Gertler

**Affiliations:** 1 Koch Institute for Integrative Cancer Research, Massachusetts Institute of Technology, Cambridge, MA 02139, USA; 2 Department of Anatomy and Structural Biology, Gruss Lipper Biophotonics Center, Albert Einstein College of Medicine, Bronx, NY 10461, USA; 3 Department of Biological Engineering, Massachusetts Institute of Technology, Cambridge, MA 02139, USA

## Abstract

During tumor progression, alternative splicing gives rise to different Mena protein isoforms. We analyzed how Mena11a, an isoform enriched in epithelia and epithelial-like cells, affects Mena-dependent regulation of actin dynamics and cell behavior. While other Mena isoforms promote actin polymerization and drive membrane protrusion, we find that Mena11a decreases actin polymerization and growth factor-stimulated membrane protrusion at lamellipodia. Ectopic Mena11a expression slows mesenchymal-like cell motility, while isoform-specific depletion of endogenous Mena11a in epithelial-like tumor cells perturbs cell:cell junctions and increases membrane protrusion and overall cell motility. Mena11a can dampen membrane protrusion and reduce actin polymerization in the absence of other Mena isoforms, indicating that it is not simply an inactive Mena isoform. We identify a phosphorylation site within 11a that is required for some Mena11a-specific functions. RNA-seq data analysis from patient cohorts demonstrates that the difference between mRNAs encoding constitutive Mena sequences and those containing the 11a exon correlates with metastasis in colorectal cancer, suggesting that 11a exon exclusion contributes to invasive phenotypes and leads to poor clinical outcomes.

Cell migration is required for physiological processes such as morphogenesis and wound healing, and is dysregulated in metastatic cancer and other diseases^1^. Cell movement requires orchestrated, dynamic remodeling of the actin cytoskeleton by an extensive repertoire of regulatory molecules that includes Ena/VASP proteins (Mena, VASP and EVL in mammals). Ena/VASP proteins regulate assembly and geometry of actin networks that, in turn, influence cell adhesion, protrusion, motility and invasion^2^,^3^. Ena/VASP proteins contribute to cell:cell and cell:matrix adhesions, and have roles in tension-regulated actin dynamics at epithelial zonula adherens^4^, epithelial morphogenetic processes such as dorsal closure in *Drosophila*^5^, and the maintenance of endothelial cell junctions^6^. An “LERER” repeat domain unique to Mena binds to the cytoplasmic tail of *α*5 integrin, and regulates *α*5β1-mediated bi-directional signaling between fibronectin and the actin cytoskeleton (Supplementary Fig. S1)^7^.

Ena/VASP proteins are processive actin polymerases that accelerate the rate of actin polymerization and delay termination of filament elongation by barbed end capping proteins^2^,^8^,^9^,^10^,^11^. They share a common structural organization, consisting of an amino-terminal EVH1 domain that mediates protein-protein interactions; a poly-proline rich central region with high-affinity binding sites for profilin; a C-terminal EVH2 domain that contains G- and F-actin binding sites and a carboxy-terminal coiled-coil that mediates homo- and hetero-tetramerization of Ena/VASP proteins^2^ (Supplementary Fig. S1). This structure allows Ena/VASP to bind profilin:G-actin complexes and transfer the actin monomer to adjacent F-actin barbed ends^11^. Ena/VASP proteins can also influence the activity of actin regulators including the Arp2/3 complex and formins^2^,^12^,^13^. Ena/VASP proteins promote filopodium formation and dynamics by direct effects on actin polymerization^14^,^15^, and through interaction with the formin-family Diaphanous/mDia2 F-actin nucleation/elongation factors^16^,^17^ and the multifunctional Eps8 and IRSp53 proteins^18^. In addition, Ena/VASP proteins play key roles in the elongation of F-actin tails induced by the intracellular pathogen *Listeria monocytogenes*^19^.

Mena is upregulated in human epithelial tumors (breast, pancreas, colon, and cervix)^20^,^21^,^22^,^23^ and in invasive rodent mammary adenocarcinoma cells collected by *in vivo* EGF-elicited chemotaxis^24^. In the MMTV-PyMT murine model of invasive breast cancer, Mena deficiency has no significant effect on carcinoma growth, but delays tumor progression and reduces invasion, intravasation, and metastatic spread of carcinoma cells^25^.

The Mena mRNA can contain one or more of 5 alternatively-included exons that produce in-frame proteins^26^,^27^,^28^; inclusion of at least some of these exons is associated with specific tumor cell phenotypes *in vivo*^3^. One isoform, Mena^INV^, is upregulated in chemotactic, invasive mammary carcinoma cells. Mena^INV^ sensitizes carcinoma cells to stimulation with certain growth factors, and increases their invasive and metastatic capability^29^,^30^,^31^. The Mena11a isoform, which includes a conserved 21 amino acid sequence between the F-actin binding and tetramerization regions in EVH2^27^ (Supplementary Fig. S1), is abundant in epithelial carcinoma cell lines and low/absent in invasive carcinoma cells as well as in cells that have undergone an epithelial-to-mesenchymal transition (EMT)^21^,^27^.

Several biomarkers that include assessment of Mena immunostaining, or that measure expression of Mena isoforms show significant correlations with clinical outcomes in breast cancer patients. For example, the risk of distant metastasis in ER+/HER2− patients correlates with TMEM (tumor microenvironment of metastasis) density, a measure of tripartite anatomical structures containing an endothelial cell, a macrophage and a carcinoma cell expressing Mena^32^. In addition, Mena^INV^ levels are significantly higher in metastatic, than in non-metastatic primary tumors, and correlate with poor clinical outcome and relapse across subtypes^33^. Survival of breast cancer patients with tumors exhibiting high overall Mena, but low Mena11a expression is significantly reduced^34^,^35^, suggesting that Mena11a expression could antagonize mechanisms associated with tumor progression, such as the acquisition of metastasis-associated phenotypes. Consistent with this possibility, Mena11a expression in mammary carcinoma cells decreases EGF-elicited 3-dimensional (3D) invasion both *in vitro* and *in vivo,* and mammary tumors formed by Mena11a-expressing cells do not metastasize efficiently^30^.

The cellular and molecular underpinnings of Mena11a-dependent phenotypes are poorly understood. Here we reveal isoform-specific and phospho-regulated roles for Mena11a that are functionally distinct from Mena in the control of actin cytoskeleton organization, cell:cell adhesion and motility in cancer cells.

## Results

### Mena11a expression in normal epithelial structures and carcinomas

Mena11a is expressed in carcinomas and epithelial-like cell lines (Supplementary Fig. S1)^21^,^27^,^36^,^37^, and forced expression of Mena11a in xenografted mammary cancer cells promotes formation of tumors with cohesive, epithelial like phenotypes^31^; however, the extent to which Mena11a is expressed in normal tissue epithelia is unknown. We compared Mena and Mena11a distribution by immunofluorescence, using antibodies that recognize all Mena isoforms (“pan-Mena”) and a Mena11a-isoform specific antibody to stain mouse and human tissues. In developing mouse E15.5 dermis and E15.5 lung, Mena11a localized to cells in the epidermis (Supplementary Fig. S1) and lung epithelium (Supplementary Fig. S1), respectively, but was excluded from surrounding pan-Mena-expressing mesenchyme; Mena11a expression was retained in adult mouse and human epithelial tissues, including mouse epidermis (Supplementary Fig. S1), mouse bronchioalveolar epithelium (Supplementary Fig. S1), and human colon epithelium (Supplementary Fig. S1), while pan-Mena signal was observed in non-epithelial cells in these same tissues. Thus, we conclude that Mena11a is enriched in normal epithelial structures *in vivo*.

**Figure 1:**
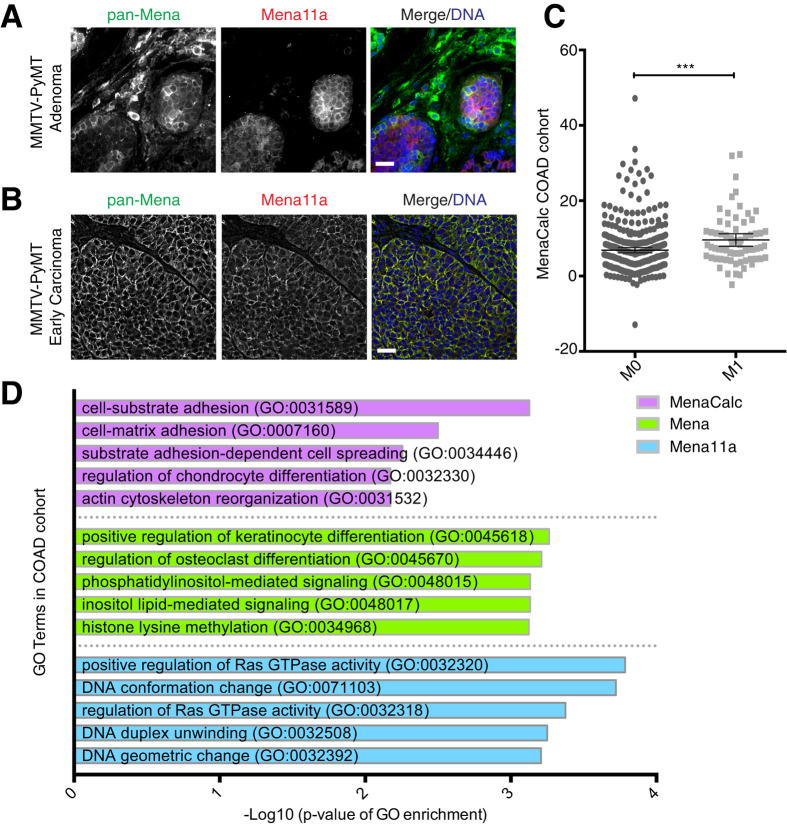
Analysis of Mena11a expression in epithelial cancers. Immunofluorescence of pan-Mena and Mena11a in primary mammary tumors from MMTV-PyMT transgenic mice at both the (**A**) adenoma and (**B**) early carcinoma stages. (**A**,**B**): DNA visualized with Hoechst staining. Scale bar, 20 μm. Images are representative of three independent experiments. (**C**) Association between metastatic stage and MenaCalc in COAD patient cohort; M0 = no evidence of distant metastasis, M1 = evidence of distant metastasis. For M0, n = 345 patients, for M1, n = 67 patients. Error bars: 95% CI. Wilcoxon rank-sum test ***p < 0.005. (**D**) GO term enrichment categories of the top 50 genes correlated with MenaCalc, Mena, and Mena11a in the COAD cohort.

Several epithelial human cancers express high levels of Mena^20^,^21^,^22^,^23^. While Mena11a mRNA and protein levels have been examined in fine needle aspirates and in clinical samples^31^,^34^,^35^, the spatial distribution of Mena11a protein during tumor progression has not been reported. We examined Mena11a expression during progression of MMTV-PyMT mouse mammary tumors. In adenomas and early carcinomas (Fig. 1A,B, respectively), pan-Mena and Mena11a had heterogeneous expression: whereas Mena11a and pan-Mena were enriched in tumor cells with epithelial-like morphology, Mena11a was excluded from the pan-Mena positive stromal cells.

MenaCalc is a biomarker that assesses the difference between normalized pan-Mena and Mena11a levels by multiplexed quantitative immunofluorescence^34^. High MenaCalc (e.g. relatively high Mena and low Mena11a) levels correlated significantly with decreased overall survival in three independent breast cancer patient cohorts^34^,^35^, although levels of either Mena or Mena11a alone did not. To investigate whether RNAseq transcriptome data from clinical samples could be used to develop a surrogate metric equivalent to MenaCalc, we acquired exon-level gene expression data from publicly available TCGA data to determine whether the abundance of mRNAs encoding constitutively included Mena exons, Mena11a, or an mRNA-based version of MenaCalc (see Methods) were associated with overall survival. We were unable to find a stable correlation between MenaCalc and overall survival in the TCGA breast cancer cohort (BRCA)^38^, likely due to the short follow-up time (of patients with >10 years follow-up, n = 55 alive, n = 73 deceased, p = 0.05 with ¼ cutoff point) in this cohort. We were also unable to detect a significant correlation between MenaCalc levels and metastasis formation in this relatively small cohort of TCGA BRCA patients.

Since Mena11a is expressed in normal human colon epithelium (Supplementary Fig. S1), and Mena is upregulated in colorectal adenocarcinomas^22^, we investigated whether MenaCalc levels correlated with overall survival or with annotated clinicopathological characteristics in the TCGA colorectal cancer cohort (COAD)^39^. While we failed to detect a significant correlation between MenaCalc and overall survival, by either Kaplan-Meier analysis or using logistic regression (again, likely because of the relatively short follow-up time and small number of patients in the cohort; >1 year follow-up, n = 110 alive, n = 33 deceased), patients with distant metastasis (M1) had, on average, significantly higher MenaCalc values compared to patients with no evidence of distant metastasis (M0) (Fig. 1C). Logistic regression analysis demonstrated that MenaCalc (coefficient = 0.349, p = 0.003), but neither Mena (coefficient = 0.176, p = 0.168), nor Mena11a (coefficient = −0.033, p = 0.808) alone, was significantly associated with metastasis in the COAD cohort. These data support the idea that MenaCalc is associated with malignant progression in at least some carcinomas.

Interestingly, gene ontology (GO) and gene set enrichment analysis (GSEA) analyses of genes whose expression levels correlated with those of Mena, Mena11a, or MenaCalc showed that a distinct set of functional annotations were enriched in the MenaCalc, but not the Mena or Mena11a correlating gene lists (Fig. 1D and Table 1). The top 50 genes correlating with MenaCalc in the COAD cohort (Supplementary Table S1), including EMT markers Zeb1 and Zeb2, were enriched in gene sets related to EMT (Table 1), and were associated with GO terms such as cell:substrate adhesion (GO:0031589) and cell:matrix adhesion (GO:0007160) (Fig. 1D). Within the top 150 genes, we find that SNAI2, or Slug, another hallmark EMT gene, correlates with MenaCalc in the COAD cohort (Supplementary Table S1); however, we did not find a strong correlation with either Snail or Twist (rho = 0.319 and 0.399, respectively). In contrast, the sets of genes correlating with expression of either Mena or Mena11a alone (Supplementary Table S1) did not show any significant enrichment for terms associated with key biological processes involved in EMT, or cancer invasion and metastasis. These findings are consistent with the idea that MenaCalc, which represents the abundance of Mena isoforms lacking the 11a exon, is more associated with pro-metastatic phenotypes than either total Mena or Mena11a levels, and provides insight into why MenaCalc, but not Mena or Mena11a levels, are associated with poor clinical outcome in appropriately powered analyses of multiple breast cancer patient cohorts^34^,^35^.

### Mena11a maintains cell-cell junctions by regulating F-actin structure

**Figure 2:**
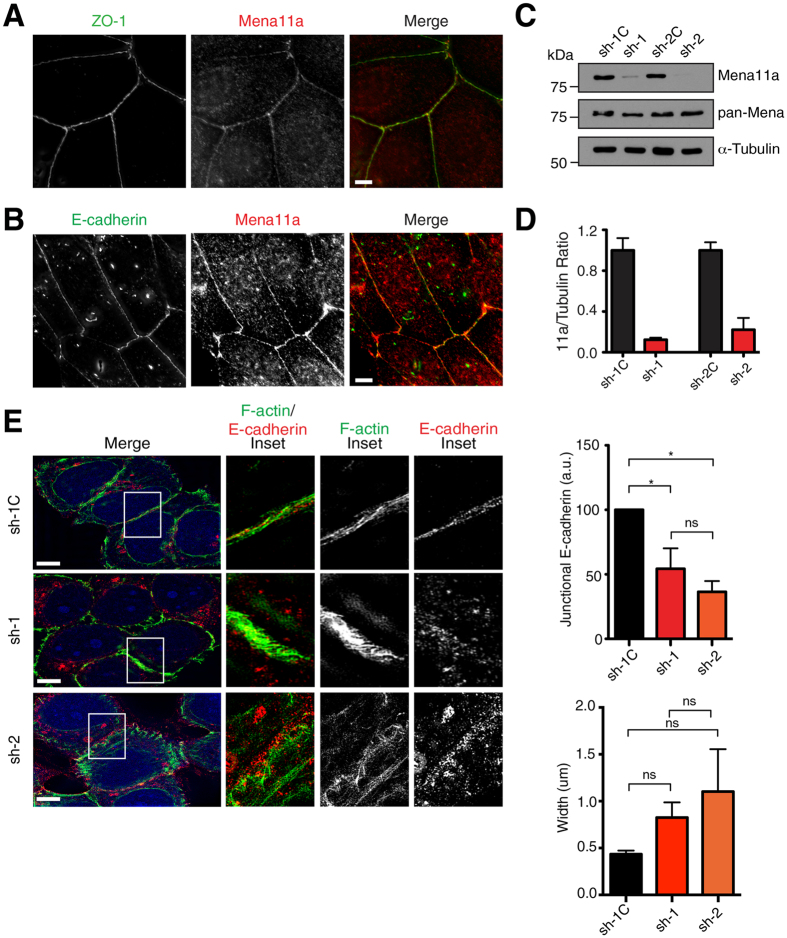
Mena11a expression maintains junctional integrity. (**A**–**E**): MCF7 cells. (**A**) Immunofluorescence showing endogenous ZO-1 and Mena11a localization. Scale bar, 10 μm. (**B**) Immunofluorescence showing endogenous E-cadherin and Mena11a localization. Scale bar, 10 μm. (**C**) Western blot analysis. Membranes probed with anti Mena11a and anti pan-Mena antibodies. *α*-Tubulin: loading control. (**D**) Quantitative analysis of relative ratio of Mena11a:*α*-Tubulin, determined by densitometry. Fold change in expression is relative to appropriate control. Error bars: SEM. Results represent triplicates. (**E**) (Left panel) 3D-SIM images showing E-cadherin localization in MCF7 cells with isoform-specific knockdown of Mena11a, using two different shRNAs (sh-1, sh-2) and control shRNAs (sh-1C). Space-filling GFP in blue indicates cells expressing Mena11a shRNAs or control shRNAs. F-actin is visualized by phalloidin labeling. Insets: 7X magnification. Scale bar, 10 μm. (Right panel, top) Quantitation of junctional E-cadherin fluorescence intensity. a.u. = arbitrary units. >30 cells analyzed. Error bars: SEM. Results represent triplicates. One-way ANOVA *p < 0.05, n.s., not significant. (Right panel, bottom) Lateral distribution of junctional E-cadherin. >30 cells analyzed. Error bars: SEM. Results represent triplicates. One-way ANOVA n.s., not significant.

Mena11a is enriched in epithelia; we find it preferentially targets to cell:cell contacts *in vivo* (Fig. 1 and Supplementary Fig. S1), and co-localizes with ZO-1 at tight junctions (Fig. 2A) as well as E-cadherin at adherens junctions (Fig. 2B) in cultured human breast cancer MCF7 cells. In addition, calcium switch experiments in primary mouse keratinocytes showed that Mena11a was recruited to nascent E-cadherin-positive adherens junctions that form upon re-addition of calcium (Supplementary Fig. S2).

Ectopic expression of Mena11a in xenografted human breast cancer cells can drive formation of tumors characterized with a cohesive, epithelial-like morphology^31^; however, such overexpression assays cannot identify specific requirements for Mena11a function in tumor cell morphology. To assess whether the 11a sequence endows Mena11a with specific functions distinct from Mena, we designed shRNAs targeting the 63 bases of the 11a insertion (sh-1, sh-2, hereafter Mena11a-KD) and paired control shRNAs (sh-1C, sh-2C, hereafter control-KD). In MCF7 cells, Mena11a shRNAs efficiently downregulated Mena11a, but did not affect protein levels of Mena lacking the 11a insertion (Fig. 2C,D and Supplementary Fig. S2).

To investigate Mena11a-isoform specific function at cell-cell contacts, we used super-resolution three dimensional structured illumination microscopy (3D-SIM) to image monolayers of MCF7 Mena11a-KD cells and control-KD cells that were stained with phalloidin, ZO-1, or E-cadherin to visualize F-actin, tight junctions (Supplementary Fig. S2), or adherens junctions (Fig. 2E), respectively. Mena11a-KD cells had reduced E-cadherin accumulation at adherens junctions, as shown by fluorescence intensity quantitation (Fig. 2E), but no difference in the lateral distribution of E-cadherin when compared to control-KD cells (Fig. 2E). Normal circumferential F-actin belts adjacent to tight-, and adherens-junctions in epithelial sheets^40^ were apparent in control-KD cells; however, in Mena11a-KD cells, the F-actin appeared to be disorganized at both tight- (Supplementary Fig. S2) and adherens-junctions (Fig. 2E). Together, these data indicate that the Mena11a isoform influences the architecture of cell:cell contacts distinctly from other Mena isoforms (whose expression is not affected by isoform-specific depletion of Mena11a), and from other Ena/VASP family members.

### Mena11a-specific depletion enhances cell migration and membrane protrusion

**Figure 3:**
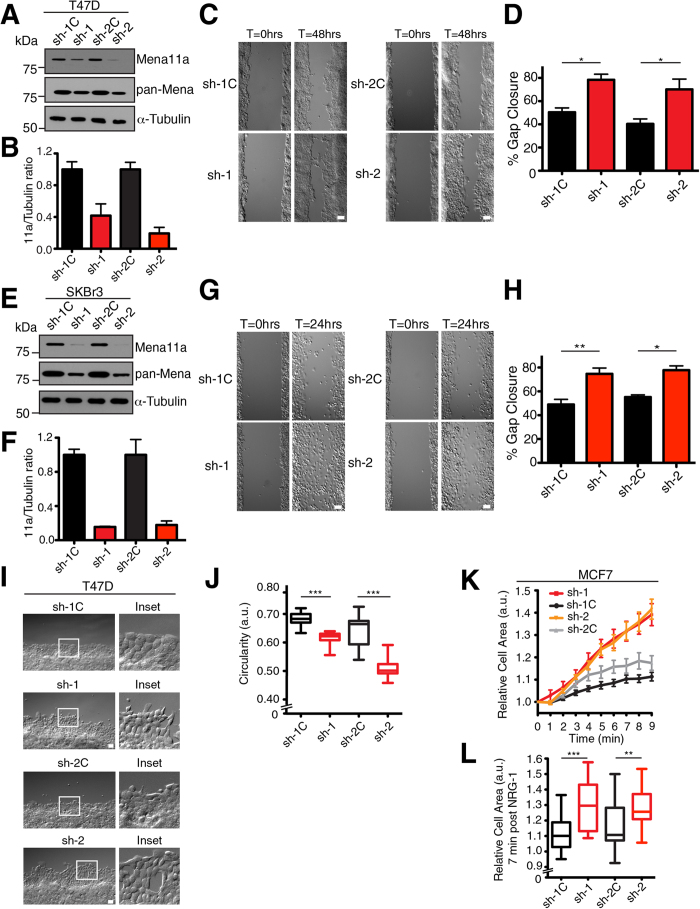
Mena11a downregulation affects migration, morphology and membrane protrusion. (**A,E**): Western blot analysis of lysates from (**A**) T47D and (**E**) SKBr3 cells with stable, isoform-specific knockdown of Mena11a using two different shRNAs (sh-1, sh-2, respectively) and control shRNAs (sh-1C, sh-2C). Membranes were probed with anti pan-Mena and anti Mena11a antibodies. α-Tubulin was used as the loading control. (**B**,**F**): Quantitative analysis of the relative ratio of Mena11a: α-Tubulin as determined by densitometry in (**B**) T47D and (**F**) SKBr3 control and Mena11a-specific knockdown cells. Fold change in expression is relative to the appropriate control. Error bars: SEM, results represent triplicates. (**C**,**D**,**I**,**J**): Wound healing assay using T47D control (sh-1C, sh-2C) and Mena11a-specific knockdown (sh-1, sh-2) cells. (**C**) DIC images of cells after 0 and 48 hours in complete media. Scale bar, 50 μm. (**D**) Percent gap closure of cells after 48 hours in complete media. (**G**,**H**): Wound healing assay using SKBr3 control (sh-1C, sh-2C) and Mena11a-specific knockdown (sh-1, sh-2) cells. (**K**,**L**): MCF7 control and Mena11a-specific knockdown cells stimulated with 100 ng/ml Neuregulin-1. Quantitative results in (**D**,**H**,**J**,**L**) represent triplicates, error bars represent SEM. Unpaired t-test, *p < 0.05, **p < 0.01, ***p < 0.005. (**G**) DIC images of cells after 0 and 24 hours in complete media. Scale bar, 50 μm. (**H**) Percent gap closure of cells after 24 hours in complete media. (**I**) Morphology of cells. DIC images of the gap’s free edge after 24 hours. Scale bar, 50 μm. Insets are 9x magnification. (**J**) Morphometric analysis of cells. DIC images of the gap’s free edge after 24 hours. Circularity = 
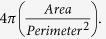
Box and whisker plots of circularity; >470 cells analyzed. (**K**) Membrane protrusion kinetics; >22 cells analyzed. (**L**) Membrane protrusion of control and Mena11a-specific knockdown cells at t = 7 minutes post stimulation; >22 cells analyzed. Cells were starved for 4 hrs prior to stimulation with 100 ng/ml NRG-1.

Previously, the effects of Mena11a on cancer cell motility were evaluated only in assays utilizing ectopic expression^30^. To study the role of endogenously expressed Mena11a on cancer cell motility, we performed wound closure assays in Mena11a-KD cells, including T47D and SKBr3 human breast cancer cells, which have >80% reduction of Mena11a protein levels (Fig. 3A,B,E,F). 24 hours after exposing a gap in a monolayer of SKBr3 control-KD cells, approximately 52% of the initially cell-free region was filled (sh-1C: 49.03% ± 4.2; sh-2C: 55.24% ± 1.8), while SKBr3 Mena11a-KD cells filled significantly larger areas (sh-1: 74.76% ± 4.8; sh-2: 77.84% ± 3.6) (Fig. 3G,H), indicating that Mena11a-depleted cells exhibited enhanced migration. T47D cells yielded similar results (Fig. 3C,D), although complete closure by the control T47D cells took longer than with SKBr3 cells. T47D control-KD cells had filled approximately 45% of the cell-free region (sh-1C: 50.41% ± 3.7; sh-2C: 40.38% ± 4.2) after 48 hours, while T47D Mena11a-KD cells filled approximately 74% of it (sh-1: 78.39% ± 4.8; sh-2: 70.20% ± 8.8) (Fig. 3C,D). Therefore, depletion of the Mena11a isoform from cells that normally express both Mena and Mena11a increased cell migration rates.

Consistent with changes in migration, we observed that the morphology of T47D Mena11a-KD cells differed from control-KD cells, specifically at the free edge of the monolayer (Fig. 3I). The circularity (perfect cell circularity = 1; decreasing values correlate with increasing cellular elongation; see Methods) of control-KD cells was 0.64–0.68 (sh-1C: 0.683; sh-2C: 0.640), as expected for cells with typical cobblestone epithelial morphology, whereas that of Mena11a-KD cells was significantly lower, 0.51–0.61 (sh-1: 0.614; sh-2: 0.509) indicating that they had a more elongated morphology (sh-1C vs sh-1 p < 0.005; sh-2C vs sh-2 p < 0.005) (Fig. 3J).

Growth factor-elicited membrane protrusion correlates with 3D migration of breast cancer cells^41^, and with dissemination and metastasis of carcinoma cells^42^. MCF7 cells respond to acute Neuregulin-1 (NRG-1) treatment by membrane protrusion^43^. Using time-lapse imaging, we found that Mena11a-KD MCF7 cells exhibited significantly increased membrane protrusion, measured by fold change in cellular area, compared to control-KD cells (Fig. 3K,L). Together, these data indicate that endogenous Mena11a reduces cell motility and attenuates growth factor elicited lamellipodium extension in epithelial breast cancer cells. These effects of Mena11a are distinct from those of Mena, which increases breast cancer cell motility and lamellipodium protrusion^29^.

### Effect of Mena11a on actin cytoskeletal organization

**Figure 4:**
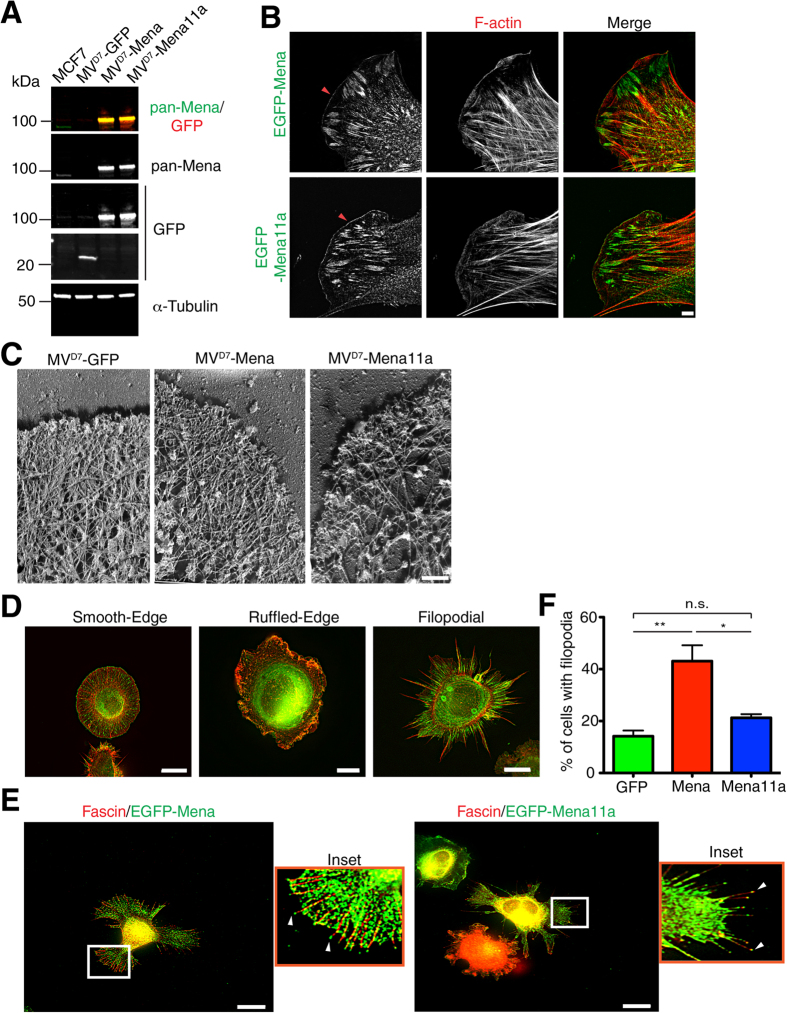
Mena11a homotetramers alter F-actin organization at the leading edge, target to tips of filopodia and decrease filopodia formation. (**A**) Western blot of lysates from MV^D7^ cells expressing GFP, Mena or Mena11a and MCF7 cells. Membranes were probed with anti pan-Mena and GFP antibodies. α-Tubulin is used as the loading control. (**B**) 3D-SIM images of EGFP-Mena in MV^D7^ EGFP-Mena cells (top) and EGFP-Mena11a in MV^D7^ EGFP-Mena11a cells (bottom). Phalloidin staining shows F-actin. Scale bar, 10 μm. Red arrowheads: Mena and Mena11a localize properly to the leading edge of lamellipodia. (**C**) Platinum replica EM of actin cytoskeleton in MV^D7^ cells expressing GFP, Mena and Mena11a, stimulated with 100 ng/ml PDGF-BB for 5 minutes. Scale bar, 250 nm. Images representative of seven independent experiments. (**D**) Immunofluorescence of the spreading assay (MV^D7^ cells plated on 20 μg/ml laminin). The three spreading phenotypes depicted are: smooth-edge (left panel), ruffled-edge (middle panel) and filopodial (right panel). F-actin visualized by phalloidin labeling. Scale bar, 10 μm. (**E**) Immunofluorescence of spreading MV^D7^ cells expressing EGFP-Mena (left panel) and EGFP-Mena11a (right panel). Fascin antibodies are used as a filopodial marker. Scale bar, 10 μm. For EGFP-Mena MV^D7^ cells, inset at 10X magnification shows Mena localization at the tips of filopodia. For EGFP-Mena11a MV^D7^ cells, inset at 11X magnification shows Mena11a localization at the tips of filopodia. (**F**) Percent of spreading MV^D7^ cells expressing GFP, Mena, and Mena11a with the filopodial phenotype. Results represent triplicates, >930 cells analyzed. Error bars represent SEM. One-way ANOVA *p < 0.05, **p < 0.01, n.s.: not significant.

The Mena11a isoform-specific depletion phenotypes at cell:cell junctions and membrane protrusions raise the possibility that Mena11a may affect actin cytoskeleton remodeling differently than Mena. We explored the contribution of Mena11a to actin cytoskeletal organization in an established cell line model used to study Ena/VASP function. MV^D7^ cells, which lack Ena/VASP proteins^44^, were used to generate a panel of cell lines expressing equivalent levels of GFP, GFP-Mena, or GFP-Mena11a (hereafter GFP, Mena and Mena11a cells) (Fig. 4A). Expression of Mena or Mena11a individually in an Ena/VASP protein-deficient background cell line simplifies the interpretation of results by excluding the potential effects of heterotetramers comprised of mixed Mena isoforms and other Ena/VASP proteins. 3D-SIM imaging revealed that Mena and Mena11a each localized to the leading edge and to focal adhesions in MV^D7^ cells (Fig. 4B); thus, Mena11a is targeted to common Ena/VASP localization sites within cells.

The known role of Ena/VASP proteins in controlling the architecture of assembling actin networks^9^ led us to compare how actin networks were assembled in MV^D7^ cells expressing Mena11a or Mena. GFP-, Mena-, and Mena11a-expressing MV^D7^ cell lines were stimulated for 180 seconds with 100 ng/ml PDGF-BB to induce lamellipodium protrusion and then fixed and used to make platinum replicas of the nascent lamellipodia. Electron microscopy was then used to examine the supramolecular organization of the actin filament networks within lamellipodia. Compared to GFP control cells, the F-actin network density did not appear to be altered grossly by Mena expression, but appeared to be substantially diminished in the lamellipodia of Mena11a expressing MV^D7^ cells (Fig. 4C).

In addition to lamellipodial protrusion, Mena (and other Ena/VASP proteins), promotes filopodia formation and elongation^14^,^15^,^16^,^17^. We analyzed the ability of Mena11a to support filopodium formation during cell spreading. MV^D7^ cells spread on laminin via three morphologically distinct spreading modes: smooth edge, ruffle edge, and filopodial; expression of either Mena or VASP in MV^D7^ cells increases the percentage of cells spreading with a filopodial phenotype^15^ (Fig. 4D). We found that when MV^D7^ cells spread on laminin, both Mena and Mena11a localized to filopodial tips (Fig. 4E). As expected, Mena expression increased the percentage of cells spreading with a filopodial phenotype. In contrast, Mena11a expression did not support efficient filopodium formation, as the percentage of cells spreading with filopodia was similar to that of control MV^D7^ cells (Fig. 4F).

### Expression of Mena11a dampens cancer cell membrane protrusion

**Figure 5:**
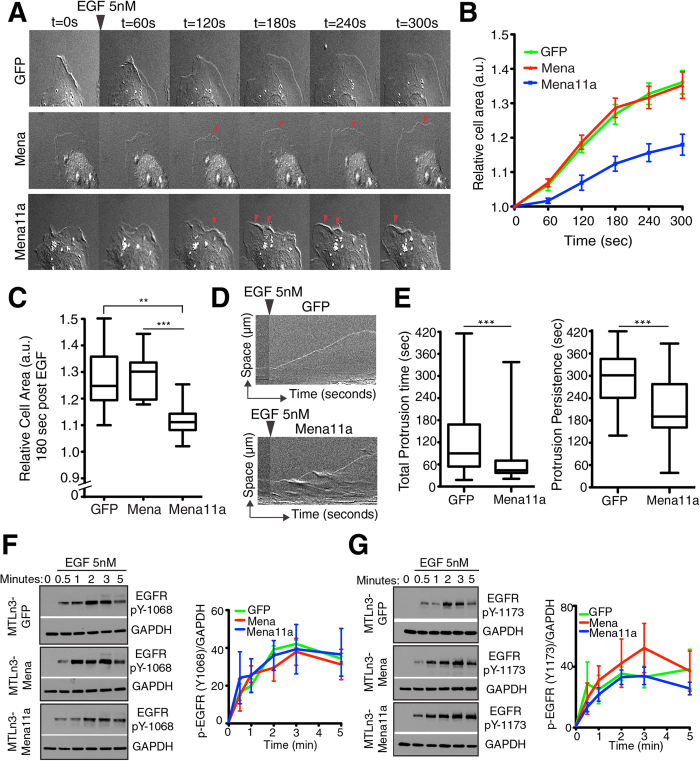
Mena11a expression dampens membrane protrusion but EGFR activation is unaffected. (**A**–**C**): MTLn3 cells stably expressing GFP, Mena and Mena11a, stimulated with 5 nM EGF. (**A**) DIC images of membrane protrusion during stimulation. Red arrowheads: lamellipodial protrusions. Protrusions are evident in Mena cells, but dampened in Mena11a cells. (**B**) Membrane protrusion kinetics of cells after EGF stimulation. Error bars: SEM. (**C**) Membrane protrusion after t = 180 seconds. Error bars: SEM. Results represent triplicates, >90 cells analyzed. One-way ANOVA **p < 0.01, ***p < 0.005. (**D**) Kymographs from time-lapse movies of MTLn3 GFP and MTLn3 GFP-Mena11a cells stimulated for 300 seconds. Kymographs demonstrate lamellipodial activity; ascending contours of edge represent protrusions, while descending ones represent withdrawals. (**E**) Box and whisker plot quantifying the time of individual protrusions (left) and protrusion persistence (right) during stimulation. Data for MTLn3 GFP cells are from 112 protrusion events, for MTLn3 GFP-Mena11a cells from 90 protrusion events. Unpaired t-test ***p < 0.005. (**F**,**G**) (Left panel): Western blot analysis of MTLn3 cells stably expressing GFP, Mena and Mena11a. Cells were starved for 4 hours, then stimulated for 0, 0.5, 1, 2, 3, and 5 minutes with 5 nM EGF. Membranes were probed with (**F**) anti-EGFR pY1068 and (**G**) anti-EGFR pY1173. GAPDH used as loading control. (Right panel): Densitometry of the relative ratio of (**F**) EGFR pY1068/GAPDH and (**G**) EGFR pY1173/GAPDH as determined by densitometry. Fold increase is over baseline (no EGF stimulation).

The effects of Mena11a expression in lamellipodia and on tumor cell behavior *in vivo*^30^,^31^ prompted us to investigate the role of Mena11a in the regulation of lamellipodial behavior in MTLn3 mammary carcinoma cells. Upon bath application of EGF, MTLn3 cells initiate membrane protrusion driven by actin assembly at free barbed ends created by cofilin-mediated severing of capped F-actin filaments^45^. In MTLn3 cells, ectopic Mena or Mena^INV^ expression potentiates membrane protrusion during bath application of EGF^29^,^30^. To test the effects of Mena11a during EGF-elicited membrane protrusion, we expressed equivalent levels of GFP-Mena11a, GFP-Mena or GFP stably by retroviral transduction of MTLn3 cells (Supplementary Fig. S3). Cells were serum-starved, stimulated with different concentrations of EGF, and membrane protrusion was imaged by time-lapse microscopy (Fig. 5A). At 0.5 nM EGF (a sub-saturating dose for EGFR), expression of Mena potentiated membrane protrusion, as expected (see refs 29,31), while expression of Mena11a had no effect (Supplementary Fig. S3). In cells treated with 5 nM EGF, which elicits maximal protrusion by control MTLn3 cells^46^, Mena expression had no additional effect (Fig. 5A–C), while Mena11a expression reduced protrusion of lamellipodia significantly (Fig. 5A–C and Supplementary Movie S1). To quantify the kinetic parameters of membrane protrusion, we compared the effects of EGF stimulation on MTLn3 cells expressing either GFP or GFP-Mena11a by analyzing kymographs generated from time-lapse imaging of their lamellipodia (Fig. 5D). Compared to the GFP controls, during EGF stimulation, Mena11a expression decreased the total time the membrane was engaged in protrusions (total protrusion time), and the duration of individual protrusion events (protrusion persistence) of lamellipodia (Fig. 5E), but did not affect protrusion velocity (Supplementary Fig. S3).

The negative effect of Mena11a on growth factor elicited protrusion could arise either from a direct effect on the actin cytoskeleton, from an effect on EGFR activation and downstream signaling, or both. Use of phospho-specific antibodies against pY-1068 or pY-1173 of EGFR (rapidly phosphorylated after EGF binds the EGFR) on western blots of cell lysates from the three MTLn3 lines at various times after EGF stimulation (Fig. 5F,G) revealed no statistically significant differences in the kinetics of EGFR auto-phosphorylation (Fig. 5F,G).

### Mena11a affects cofilin- and Arp2/3-mediated barbed end formation

As Mena11a had no clear effect on EGFR activation upon saturating ligand stimulation, we focused on the effects of Mena11a on EGF-elicited actin assembly. Initially, to exclude a general Mena11a-dependent effect on targeting of actin regulatory proteins to the membrane, we stained the EGF-stimulated MTLn3 cell lines for Lamellipodin (Lpd), an Ena/VASP binding protein that is involved in lamellipodial dynamics, cell migration, and regulation of the WAVE complex^47^,^48^. No differences in Lpd localization at the lamellipodium leading edge were observed among the different cell lines (Supplementary Fig. S3).

**Figure 6:**
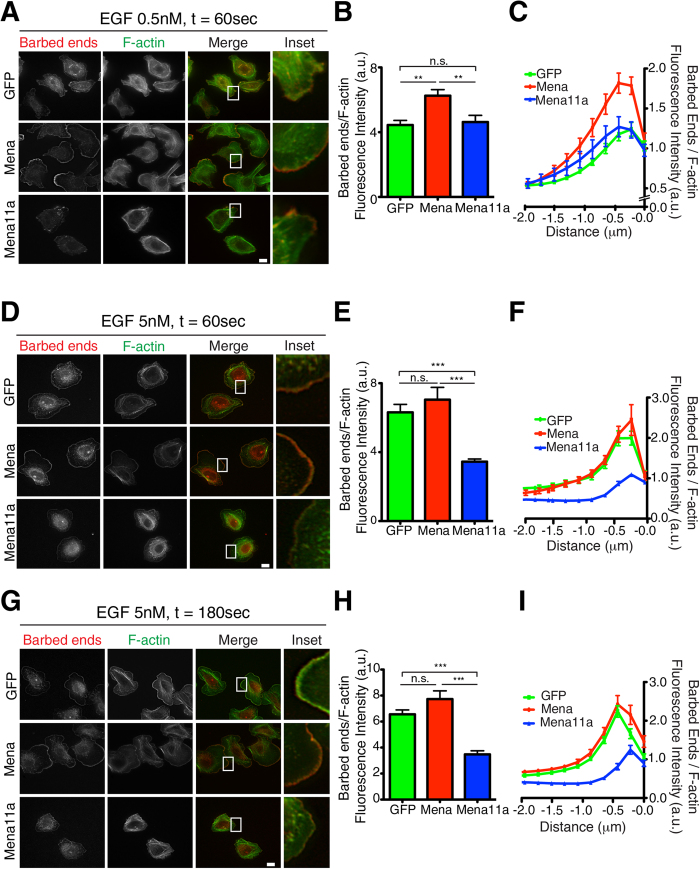
Mena11a expression decreases G-actin incorporation to F-actin barbed ends at the leading edge. All experiments done on MTLn3 cells stably expressing GFP, Mena and Mena11a. (**A**,**D**,**G**): Barbed end incorporation after stimulation with (**A**) 0.5 nM EGF for 60 seconds, (**D**) 5 nM EGF for 60 seconds, and (**G**) 5 nM EGF for 180 seconds. Barbed ends and F-actin visualized with rhodamine-G-actin and phalloidin labeling, respectively. Scale bar, 10 μm. Insets at (**A**) 27X, (**D**) 31X, and (**G**) 25X magnification show barbed end distribution at the leading edge. (**B**,**E**,**H**): Quantification of relative number of barbed ends at leading edge, after stimulation with (**B**) 0.5 nM EGF for 60 seconds, (**E**) 5 nM EGF for 60 seconds, and (**H**) 5 nM EGF for 180 seconds. Error bars: SEM. Results represent triplicates, >30 cells analyzed for (**B**,**E**) >50 cells for (**H**). One-way ANOVA **p < 0.01, ***p < 0.005, n.s not significant. (**C**,**F**,**I**): Normalized pixel intensities of relative number of barbed ends, plotted as a function of distance from the cell edge (mean ± SEM), after stimulation with (**C**) 0.5 nM EGF for 60 seconds, (**F**) 5 nM EGF for 60 seconds, and (**I**) 5 nM EGF for 180 seconds.

The abundance of actin filaments with free barbed ends correlates directly with EGF-stimulated membrane protrusion in carcinoma cells^46^. In MTLn3 cells, EGF elicited lamellipod extension depends initially on cofilin-generated actin filament barbed end formation, which peaks at 60 s, and then on Arp2/3 dependent nucleation of F-actin branches, which peaks at 180 s after stimulation^49^,^50^. Previously, we found that upon EGF stimulation, Mena is recruited to nascent lamellipodia within 30 s (preceding Arp2/3 accumulation) and potentiates barbed end formation within 60 s^29^. We measured the relative numbers of free barbed ends after stimulation with different EGF concentrations by comparing incorporation of labeled G-actin at the leading edge of permeabilized cells^51^. After 60 s of stimulation with 0.5 nM EGF, Mena expressing cells exhibited significantly greater free barbed end labeling compared to Mena11a expressing and control cells (Fig. 6A–C). In cells treated with 5 nM EGF, barbed end levels of Mena11a were significantly lower than both control and Mena expressing MTLn3 cells after 60 s (Fig. 6D,F) and 180 s (Fig. 6G–I). Hence, Mena11a reduces both cofilin-dependent (at 60 s) and Arp2/3-dependent (at 180 s) free barbed end abundance within lamellipodia of carcinoma cells stimulated with a saturating EGF concentration.

### A phosphorylation site within Mena11a regulates its activity

**Figure 7:**
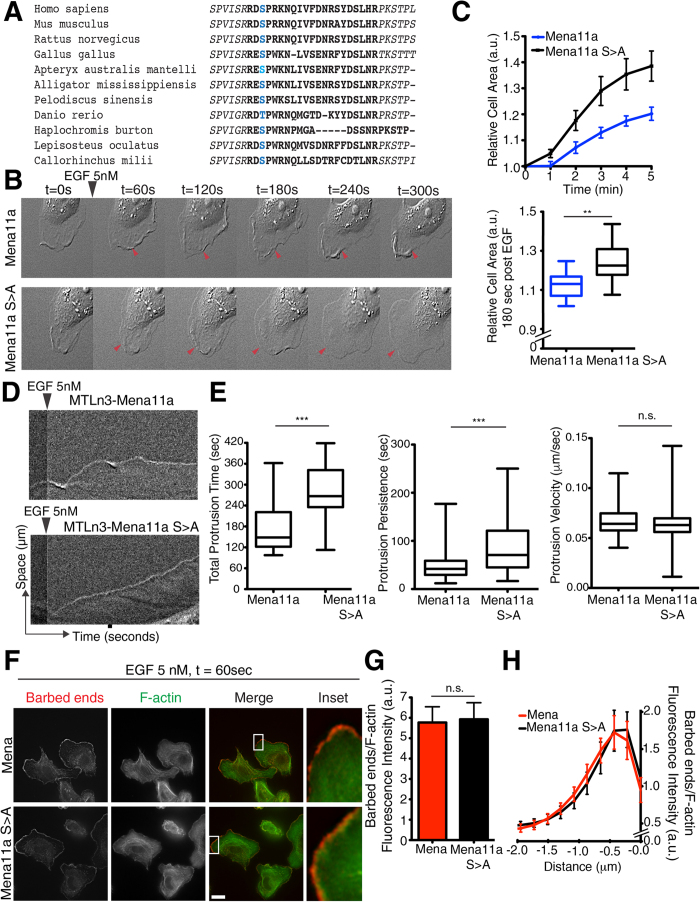
A serine phosphorylation site in Mena11a regulates its function. (**A**) Alignment of Mena11a protein sequences across species. Blue: conserved serine 3 in the 11a insertion sequence. (**B**–**H**): MTLn3 cells, stably expressing Mena11a and Mena11a S > A mutant stimulated with 5 nM EGF. (**B**) DIC images of membrane protrusion during stimulation. Arrowheads indicate dampened membrane protrusions in Mena11a cells, lamellipodial protrusions in Mena11a S > A mutant cells. (**C**) (Top): Membrane protrusion kinetics of >195 cells. (Bottom): At t = 180 seconds after stimulation. Unpaired t-test **p < 0.01. (**D**) Kymographs from time-lapse DIC movies of MTLn3 cells stably expressing Mena11a and the Mena11a S > A mutant, and stimulated with 5 nM EGF for 300 seconds. Kymographs demonstrate lamellipodial activity; ascending contours of edge represent protrusion events, descending contours of edge represent withdrawal events. (**E**) Box and whisker plots of time, protrusion persistence, and velocity of individual protrusion events for MTLn3 cells stably expressing Mena11a and the Mena11a S > A mutant while being stimulated with 5 nM EGF. Center-line of box indicates the median, top indicates the 75^th^ quartile, bottom indicates the 25^th^ quartile. Whiskers represent 10^th^ and 90^th^ percentiles. Data from 90 events of protrusion; Unpaired t-test ***p < 0.005, n.s.: not significant. (**F**) Barbed end incorporation in cells after stimulation for 60 seconds. Barbed ends and F-actin visualized with rhodamine-G-actin and phalloidin labeling, respectively. Scale bar, 10 μm. Insets at 38X magnification show barbed end distribution at leading edge. (**G**) Quantification of relative number of barbed ends at leading edge after stimulation for 60 seconds in >20 cells. Unpaired t-test, n.s. not significant. (**H**) Normalized pixel intensities of relative number of barbed ends plotted as a function of distance from the cell edge (mean ± SEM) of cells after stimulation for 60 seconds. (**C**,**E**,**G**): Results done in triplicates, error bars represent SEM.

The Mena11a insertion sequence harbors several putative phosphorylation sites, which are in proximity to the actin binding sites within the EVH2 domain, suggesting that Mena11a-specific phosphorylation might alter its effects on actin polymerization. While isoform-specific phosphorylation of Mena11a, detected by 2D electrophoresis, has been reported^27^, the specific phosphorylation site(s) within the 11a sequence have not been determined. We immunoprecipitated GFP-Mena11a from engineered MTLn3 cells 60 s after 5 nM EGF stimulation (Supplementary Fig. S4), and, using mass spectrometry, identified a unique serine phosphorylation site (hereafter serine 3) within the 21 amino acid of the 11a sequence (Supplementary Fig. S4, demarcated in blue). Alignment of the Mena11a protein sequences from several vertebrate species revealed conservation of this serine and the residues flanking the site (Fig. 7A). To study the contribution of S3 phosphorylation to Mena11a function, we generated MTLn3 cells expressing a non-phosphorylatable S3 >A Mena11a mutant (Mena11aS > A; Supplementary Figs S3 and S4) and a phosphomimetic S3 > D Mena mutant (Mena11aS > D; Supplementary Fig. S4). As before, after stimulation with 5 nM EGF, cells expressing Mena demonstrated a clear increase in membrane protrusion relative to cells expressing Mena11a (Fig. 6D–I, Supplementary Fig. S4). Interestingly, Mena11aS > A expression induced membrane protrusions that were more similar to Mena than Mena11a, both in terms of area of increase and morphology: Mena11aS > A lamellipodia protruded as flat sheets, whereas Mena11a cells displayed failed protrusions (Fig. 7B,C, Supplementary Movie S2). Kymograph analysis showed that, compared to Mena11a cells, lamellipodia in the Mena11aS > A cells exhibited significant increases in the total time that the membrane was engaged in protrusions (total protrusion time), and in the duration of individual protrusion events (protrusion persistence) (Fig. 7D,E). No significant differences in protrusion velocity were observed between the Mena11aS > A and Mena11a cells (Fig 7E). After acute stimulation with 5 nM EGF, Mena11aS > D expressing cells displayed failed protrusions similar to Mena11a cells (Supplementary Fig. S4), suggesting that S3 > D effectively mimics the effect of S3 phosphorylation on Mena11a function during growth factor stimulated protrusion. Together, these data support a model in which phosphorylation of Mena11a-S3 is necessary to dampen growth-factor elicited protrusions.

We next compared the effects of Mena11aS > A and Mena on EGF-elicited barbed end abundance. In contrast to cells expressing Mena11a (Fig. 6D–F), 5 nM EGF stimulation of cells expressing Mena11aS > A lead to increases in free barbed end abundance similar to those observed in Mena expressing cells (Fig. 7G,H). Thus, Mena11aS > A partially mimics Mena function in lamellipodium protrusion and F-actin free barbed end formation, demonstrating that phosphorylation is required for Mena11a specific functions.

## Discussion

This is the first study directly comparing Mena to Mena11a function in detail. We find that Mena11a is not simply an attenuated or less active form of Mena, but is endowed with unique and distinct functions that can be observed in the absence of Mena or other Ena/VASP proteins. Mena11a decreases the abundance of free barbed ends and the F-actin network density in lamellipodia when expressed in MV^D7^ cells, which are otherwise Ena/VASP-deficient. Mena11a also fails to promote filopodium formation in MV^D7^ spreading cells, a process typically enhanced by, and often dependent upon Ena/VASP proteins^14^,^15^,^16^,^17^.

In addition to its unique functions, Mena11a also acts antagonistically to Mena in cells expressing both isoforms, potentially by forming mixed Mena/Mena11a heterotetramers. Using both Mena11a isoform-specific depletion and ectopic expression approaches, we found that in cells with endogenous Mena expression, Mena11a co-expression reduces the levels of F-actin barbed ends at the leading edge of lamellipodia and impairs lamellipodial protrusion and stability.

Other Mena isoforms, particularly the invasion-specific Mena^INV^ isoform enhance growth factor elicited lamellipodial protrusion^29^,^52^. In contrast, dampening of elicited lamellipodial protrusion by Mena11a likely arises as a consequence of reduced actin assembly rather than dysregulated signal transduction, as acute EGF-elicited EGFR activation and downstream signaling were unaffected in Mena11a-expressing cells. Furthermore, our findings that MCF7 cells with a 11a-specific depletion exhibit increased NRG-elicited protrusion responses suggest that Mena11a is not absolutely required for signaling through the Her2/3 pathway, which mediates NRG elicited responses, as has been proposed^53^.

Consistent with its enriched expression in epithelia within normal tissues, Mena11a contributes to epithelial phenotype: in epithelial-like breast cancer cells, Mena11a localizes to both tight- and adherens- junctions. Mena11a-specific knockdown perturbs E-cadherin distribution at cell:cell junctions, increases migration and membrane protrusion of MCF7 cells, and causes T47D cells to polarize and elongate, disrupting their normal epithelial cobble stone-like morphology. These data suggest a role for Mena11a in regulating the architecture and behavior of epithelial cells by supporting organized cell-cell contacts and a low migratory phenotype.

During EMT, expression of Mena11a, but not Mena, is down regulated by changes in alternative splicing programs during this complex phenotypic transition^37^. The epithelial-specific splicing factors ESRP1 and ESRP2 regulate exon inclusion in Mena11a and other genes involved in cell migration and expression of EMT phenotypes^36^,^54^,^55^,^56^. The pivotal role of alternative splicing programs during EMT and tumor progression is highlighted by several studies in which perturbation of the levels of ESRP1, ESRP2 or of both affected EMT-like phenotypes^28^,^36^,^37^,^54^,^55^.

Mena11a isoform-specific function is regulated by phosphorylation of Mena11a-S3. We speculate Mena11a-pS3 attenuates Mena-dependent effects on actin polymerization by adding negative charge in proximity to the actin binding sites in the EVH2 domain, thus allowing specific kinase(s) to selectively regulate Mena11a. Mena11a-S3 is contained within an evolutionarily-conserved sequence that matches consensus phosphorylation motifs for both cAMP-dependent kinase PKA and for CDK5^57^, which have roles in regulating actin dynamics^58^,^59^. The role of these and other kinases in regulating Mena11a function is a subject for further investigation; however, the presence of two additional PKA phosphorylation sites^60^ in the constitutive sequences contained in Mena complicates identification of Mena11a-specific kinases. Interestingly EVL, a Mena paralog, is also alternatively spliced to include a 21 amino acid insertion at an equivalent site between the F-actin binding and tetramerization sites. Although they lack sequence similarity, both inserted sequences are phosphorylated, raising the intriguing possibility that alternative splicing produces discrete pools of Ena/VASP proteins that are differentially regulated by different kinases.

To extend our understanding of Mena11a relevance to human cancer, we analyzed transcriptome data from multiple cancer cohorts in the Cancer Genome Atlas. While neither Mena nor Mena11a expression alone correlated with clinicopathological features of patients, an mRNA-based MenaCalc metric showed significant correlation with metastasis in colorectal adenocarcinoma patients. While transcriptomic data is not always a suitable surrogate for protein expression, the current results are consistent with previous work demonstrating a significant correlation between protein-based MenaCalc assessment and poor clinical outcome^34^,^35^. A caveat to the mRNA-based MenaCalc metric is potential stromal contamination in patient samples; in comparison, the protein-based MenaCalc metric uses cytokeratin staining to distinguish tumor and stromal cells^34^,^35^. However, the quantitative measurements for the mRNA-based MenaCalc metric bypass issues arising from signal comparison between two different primary antibodies used in the protein-based MenaCalc metric. Further analysis is necessary to better understand the specificity and sensitivity of both metrics.

In conclusion, our findings help explain why Mena11a expression promotes an epithelial phenotype and drives formation of tumors with a highly cohesive, well-differentiated appearance^31^. We propose that Mena11a expression diminishes the capacity of cancer cells to acquire aggressive, invasive phenotypes needed for metastatic progression.

## Methods

For additional details, see the SI Materials and Methods.

### Tissues

All experiments involving mice were carried out in accordance with guidelines and protocols (Protocol #1113-099-16) approved by the Committee on Animal Care, Division of Comparative Medicine at the Massachusetts Institute of Technology (MIT). All facilities are fully accredited by the AAALAC (Animal Welfare Assurance, A-3125) and meet NIH standards as set forth in the “Guide for Care and Use of Laboratory Animals” (DHHS). E15.5 dermis, E15.5 lung, adult epidermis and adult bronchioalveolar epithelium were obtained from Swiss Webster mice. Mice were sacrificed at different embryonic and adult ages, and dissected immediately. Tumors were obtained from MMTV-PyMT mice with an FVB genetic background (kindly provided by Patrick Stern and John Lamar from the Hynes laboratory at the Koch Institute, MIT, and Evanthia Roussos from the Condeelis laboratory at the Albert Einstein College of Medicine). MMTV-PyMT mice were sacrificed at 4 months. All tissues were fixed in 3.7% buffered formalin, processed and embedded in paraffin. Human colon sections were kindly provided by Daniel Chung from Massachusetts General Hospital.

### Mena11a knockdown strategy

shRNAs for Mena11a knockdown and controls were designed using (http://euphrates.mit.edu/cgi-bin/shRNA/index.pl, Hemann lab, Koch Institute, MIT). 97-mer oligos (Invitrogen) were PCR amplified with primers having EcoRI/XhoI sites and cloned into the pMSCV-LTR-miR30-SV40-GFP (MLS) vector (kindly provided by Michael Hemann, Koch Institute, MIT). Oligo sequences are reported in the SI Materials and Methods.

### Wound Closure assay

Control and knockdown cells were plated in silicone inserts with a defined cell-free gap in the same 8 well slide (Ibidi) and processed concurrently. To avoid effects related to cell proliferation, we treated cells with 5 mg/ml of mitomycin-C (Sigma), a proliferation inhibitor, 30 minutes prior to the start of the assay. DIC imaging was performed with a 10X DIC objective. Gap area was quantified after 24 (SkBr3 cells) or 48 (T47D cells) hours by manual tracing with ImageJ.

### Membrane protrusion assays

MTLn3 cells were starved for 4 hours in L15 medium (Gibco) supplemented with 0.35% BSA. Cells were stimulated with a bath application of either 0.5 nM or 5 nM EGF at 37 °C. For MV^D7^ cells and MCF7 sh11a control and knockdown cells, cells were starved as above, but stimulated with 100 ng/ml of PDGF-BB or 100 ng/ml of NRG-1 at 37 °C, respectively. DIC time-lapse movies were recorded for 5 minutes with 10 second intervals after addition of EGF, and 10 minutes with 10 second intervals, after addition of PDGF-BB or NRG-1. For MTLn3 and MCF7 cells, area fold change was quantified by cell tracing using ImageJ software. Area measurements of each cell were standardized to area at time = 0, averaged, and plotted over time after EGF or NRG-1 stimulation.

### Barbed ends assay

Barbed ends assay was performed as described^6^ with some modifications. Additional methods are included in SI Materials and Methods. The ratio of barbed end intensity to phalloidin intensity along the edge was quantified as described in SI Materials and Methods.

### Statistical analysis

Statistical differences between two conditions were determined using Student’s Unpaired t test. For multiple conditions, means were compared by analysis of variance (ANOVA). All data found to be significant (p < 0.05) by ANOVA were compared with Tukey’s honestly significant difference *post hoc* test. For box and whiskers plots, center line of box indicates the median, top indicates 75^th^ quartile, bottom indicates 25^th^ quartile; whiskers represent 90^th^ and 10^th^ percentiles.

## Additional Information

**How to cite this article**: Balsamo, M. *et al*. The alternatively-included 11a sequence modifies the effects of Mena on actin cytoskeletal organization and cell behavior. *Sci. Rep.* **6**, 35298; doi: 10.1038/srep35298 (2016).

## Supplementary Material

Supplementary Information

Supplementary Movie S1

Supplementary Movie S2

## Figures and Tables

**Table 1:** GSEA of top 50 genes correlating with MenaCalc, ENAH (Mena), and Mena11a in COAD cohort.

**MenaCalc**		
*Gene Set Name*	*# of Genes in Overlap (k)/# Genes in Gene Set (K)*	*FDR q-value*
HALLMARK_EPITHELIAL_MESENCHYMAL_TRANSITION	6/200	1.88E-04
**Mena**		
*Gene Set Name*	*# of Genes in Overlap (k)/# Genes in Gene Set (K)*	*FDR q-value*
BIOPOLYMER_METABOLIC_PROCESS	12/1684	3.26E-04
REACTOME_YAP1_AND_WWTR1_TAZ_STIMULATED_GENE_EXPRESSION	3/24	2.50E-03
REACTOME_DEVELOPMENTAL_BIOLOGY	6/396	2.67E-03
REACTOME_PPARA_ACTIVATES_GENE_EXPRESSION	4/104	2.67E-03
REACTOME_CIRCADIAN_CLOCK	3/53	1.13E-02
**Mena11a**		
*Gene Set Name*	*# of Genes in Overlap (k)/# Genes in Gene Set (K)*	*FDR q-value*
BIOPOLYMER_METABOLIC_PROCESS	12/1684	4.14E-04
RNA_METABOLIC_PROCESS	7/841	2.77E-02
TRANSCRIPTION_DNA_DEPENDENT	6/636	2.77E-02
RNA_BIOSYNTHETIC_PROCESS	6/638	2.77E-02
BIOPOLYMER_MODIFICATION	6/650	2.77E-02
