# Supplementary material for: The alternatively-included 11a sequence modifies the effects of Mena on actin cytoskeletal organization and cell behavior
Source: Sci Rep. 2016 Oct 17;6:35298. doi: 10.1038/srep35298 (PMC5066228; doi:10.1038/srep35298)
Supplement: Supplementary Information [file srep35298-s1.pdf]

Title: **The alternatively-included 11a sequence modifies the effects of Mena on actin cytoskeletal organization and cell behavior.**

Authors: Michele Balsamo[a,1], Chandrani Mondal[a,1], Guillaume Carmona[a], Leslie M. McClain[a], Daisy N. Riquelme[a], Jenny Tadros[a], Duan Ma[a], Eliza Vasile[a], John S. Condeelis[b], Douglas A. Lauffenburger[c], and Frank B. Gertler[a,2]

Supplementary Methods

Cell lines

Human cancer cell lines (MCF7, T47D, SkBr3, BT474, MDA-MB-231) and HEK 293 were obtained from ATCC and maintained in DMEM supplemented with 10% Fetal Bovine Serum (FBS, Hyclone), L-glutamine, and antibiotics (penicillin/streptomycin; Invitrogen). MTLn3 cells were maintained in alpha-MEM supplemented with 5% FBS, L-glutamine, and antibiotics (penicillin/streptomycin; Invitrogen). Adherent monolayer cultures were incubated at 37°C in 5% CO_2 and 95% air. MV^{D7}, Ena/VASP-deficient mouse embryonic fibroblastic cells were isolated as described [1], and cultured at 32°C in Immorto medium [high-glucose Dulbecco's modified Eagle's with 15% FBS, penicillin/streptomycin, L-glutamine, and 50 U/mL recombinant mouse IFN-γ (Invitrogen)]. To isolate mouse primary keratinocytes, skins from neonatal Swiss Webster mice were incubated overnight at 4°C with 2 U/ml Dispase I and II (Roche). The next day, epidermis was separated from dermis, minced, incubated for 10-20 minutes in 0.25% Trypsin (Gibco) at 37°C and passed through 45 μm cell strainers to obtain a single cell suspension of keratinocytes. Cells were maintained in calcium free S-MEM (Invitrogen), supplemented with 4% chelated FBS, 0.05 mM $CaCl_2$, 0.4 μg/ml hydrocortisone (Sigma Aldrich), 5 μg/ml bovine insulin (Sigma Aldrich), 10 ng/ml recombinant human epidermal growth factor (Invitrogen), 10^{-9} M cholera toxin (ICN), $2x10^{-9}$ M 3,3',5-triiodo-L-thyronine (T3) (Sigma Aldrich), 100 U/ml penicillin, 100 μg/ml streptomycin, 2mM L-glutamine and 0,1 gr/l $MgSO_4$. For calcium switch experiments, the calcium concentration in the culture media was raised to 1.8 mM because steady state growth conditions of the primary keratinocytes did not contain sufficient Ca2+ (0.05 mM) to support junction formation. Cell lines were tested routinely for Mycoplasma contamination by using a commercial kit (MycoAlert, Lonza).

Molecular cloning

EGFP-Mena splice isoforms were subcloned into the retroviral vector packaging Murine stem cell virus-EGFP using standard techniques. EGFP-Mena11aS>A mutant was generated using mutagenic polymerase chain reaction (PCR) primers (Stratagene) and confirmed by sequencing.

shRNAs were designed with the following web tool: http://euphrates.mit.edu/cgi-bin/shRNA/index.pl (Hemann laboratory, Koch Institute-MIT). 97-mer oligos were synthesized by Invitrogen, PCR-amplified with primers having EcoRI/XhoI sites, and cloned in the vector pMSCV-LTR-miR30-SV40-GFP (MLS) (kindly provided by Michael Hemann, Koch Institute-MIT).

sh-1
TGCTGTTGACAGTGAGCG*CATGATTCATTACACAGACCAA*TAGTGAAGCCACAGATGTA*TTGGTCTGTGTAATGAATCATA*TGCCTACTGCCTCGGA

sh-1C
TGCTGTTGACAGTGAGCG*AATGATTCCTTAAACAGCCCAA*TAGTGAAGCCACAGATGTA*TTGGGCTGTTTAAGGAATCATG*TGCCTACTGCCTCGGA

sh-2
TGCTGTTGACAGTGAGCG*CAACAGGTCCTATGATTCATTA*TAGTGAAGCCACAGATGTA*TAATGAATCATAGGACCTGTTA*TGCCTACTGCCTCGGA

sh2-C
TGCTGTTGACAGTGAGCG*AAACAGGTCATAGGATTAATTA*TAGTGAAGCCACAGATGTA*TAATTAATCCTATGACCTGTTC*TGCCTACTGCCTCGGA

Retroviral packaging, infection and fluorescence-activated cell sorting

Retroviral packaging, infection, and fluorescence-activated cell sorting were performed as previously described [1]. Briefly, retroviral plasmids were transiently transfected into HEK 293 Phoenix cells with pCL-Eco helper plasmids (for rodent cells) or plasmids containing VSV-g and GAG-Pol cDNA (for human cells); supernatant was collected after 48 hours. MV^{D7}, MTLn3, MCF7, T47D, SKBr3 and mouse primary keratinocytes were infected with virus for 24 hours in the presence of 8 mg/ml polybrene (Invitrogen) and cultured to 90% confluence, trypsinized, and fluorescence-activated cell sorting (FACS) in PBS/5% FCS. MV^{D7} and MTLn3 cells expressing EGFP-Mena isoforms were FACS-sorted to a level of expression similar to the endogenous expression of Mena in mouse embryonic fibroblasts or as described [2].

Antibodies, fluorescent probes and growth factors for cell treatment

The rabbit polyclonal anti-Mena11a [3], mouse monoclonal anti pan-Mena [4] and rabbit polyclonal anti Lamellipodin (Lpd) [5] antibodies were generated in our laboratory. Commercially available antibodies are: rabbit polyclonal anti-ZO-1 (Sigma, dilution 1:250), mouse monoclonal anti-E-Cadherin (BD, dilution 1:1000), rabbit polyclonal anti-p34Arc (Millipore, dilution 1:100), chicken IgY anti-GFP (Aves labs, dilution 1:500), rabbit polyclonal anti-GFP (BD biosciences, dilution 1:5000), mouse monoclonal anti-GFP (Clontech, dilution 1:10000), mouse monoclonal anti-Fascin (Dako, dilution 1:50), mouse monoclonal anti-Tubulin (BD biosciences, dilution 1:5000), rabbit polyclonal anti-GAPDH (Cell Signaling Technology, dilution 1:1000), rabbit polyclonal anti-EGFR pY1068 (Cell Signaling Technology, dilution 1:1000), rabbit monoclonal anti-EGFR pY1173 (Epitomics, dilution 1:1000). CF405-Phalloidin was purchased (Biotium) and diluted 1:50. Phalloidin, Alexa488 phalloidin, Alexa594 phalloidin (used 1:250 dilution) and Hoechst 33342 (used 10 μg/ml) were from Invitrogen. Mouse recombinant Epidermal Growth Factor (EGF) was from Invitrogen. Neuregulin-1 (NRG-1 β1) and Platelet Derived Growth Factor-BB (PDGF-BB) were from Peprotech. Concentrations of growth factors are indicated in figure legends.

Western blots

Cells were lysed in NP-40 buffer (1% NP-40, 150 mM NaCl, and 50 mM Tris, pH 8.0) containing protease inhibitors (Complete tablets; Roche) and phosphatase inhibitors (1 mM sodium orthovanadate, 50 mM sodium fluoride, 40 mM beta-glycerophosphate, 15 mM sodium pyrophosphate). Protein extracts were run on 8% SDS-PAGE gels, transferred to PVDF membranes (Millipore), blocked in 5% milk in TBST for 1 hour at room temperature and probed with antibodies indicated in figures and legends. Mouse and rabbit affinity purified HRP-conjugated secondaries (diluted 1:5000) were from Jackson Immunoresearch. PVDF membranes were developed with ECL reagents (GE).

For the Western blots in Figures S3, and S4, protein extracts were run on 8% SDS-PAGE gels, transferred on nitrocellulose membranes (Biorad), blocked in Licor blocking buffer for 1 hour at room temperature and probed with antibodies diluted in Licor blocking buffer as indicated in figures and legends. Mouse and rabbit affinity purified

680 and 800 fluorescently conjugated-secondary antibodies (diluted 1:10000) were from Licor. Membranes were scanned using a Licor Odyssey infrared imaging system (Licor).

G-actin purification and labeling

G-actin was extracted from rabbit muscle acetone powder and gel-filtered over a Superdex-200 gel filtration column, using standard techniques. Gel filtered G-actin was polymerized to F-actin in F-actin buffer (1 mM ATP, 5 mM $MgCl_2$, 50mM KCl, 50mM Tris/HCl, pH 8.0) and labeled with Rhodamine-X succinimidyl ester (Invitrogen) following manufacturer's instructions. F-actin was depolymerized in G-actin buffer (0.2 mM ATP, 0.5 mM DTT, 0.2 mM $CaCl_2$, 2 mM Tris/HCl, pH 8.0) to G-actin, and passed through PD-10 columns (GE Healthcare) to get rid of free rhodamine.

Barbed ends assay

MTLn3 cells were starved for 4 hours in L15 medium supplemented with 0.35% BSA. For stimulation, cells were treated with bath application of 0.5 nM or 5 nM EGF at 37°C, and 60 or 180 seconds later were permeabilized with 0.125 mg/ml saponin (Sigma) in the presence of 0.5 mM rhodamine–conjugated G-actin. After 1 minute of labeling, samples were fixed in 0.5% glutaraldehyde in cytoskeleton buffer, permeabilized with 0.5% Triton X-100 in cytoskeleton buffer, quenched in 100mM Na-Borohydride in PBS, and blocked in the presence of CF405–phalloidin (Biotium). Images were taken with a deconvolution microscope. The ratio of barbed end intensity to phalloidin intensity along the edge was quantified as described in Image Quantification below.

Kymography

DIC time-lapse sequence movies of MTLn3 cells were 5 minutes long; frames were taken every 3 seconds with a 40X DIC oil immersion objective. Kymographs were produced and analyzed using Metamorph or ImageJ. Kymographs were generated along 1-pixel-wide line regions oriented along individual protrusions. For quantitative analysis, straight lines were drawn on kymographs from the beginning to the end of

individual protrusion events, and slopes were used to calculate velocities; line projections along the x-axis (time) were used to calculate the persistence of protrusions. The protrusion time is the total time that the membrane is engaged in a protrusion, over the time of imaging.

Platinum replica electron microscopy

Platinum Replica Electron Microscopy was performed as described [6]. MV^{D7} cells were cultured on coverslips and immediately extracted with 1% Triton X-100 in PEM buffer (100 mM PIPES, pH 6.8, 1 mM EGTA, 1 mM $MgCl_2$) containing 10 μM phalloidin, 0.2% glutaraldehyde, and 4.2% sucrose as an osmotic buffer. Coverslips were washed with PEM containing 1 μM phalloidin, and 1% sucrose, fixed in 0.1 M Na-cacodylate buffer (pH 7.3), 2% glutaraldehyde, 1% sucrose, and processed for electron microscopy. Images were captured on film using a TEM JEOL 200CX. Films were scanned and an unsharp mask filter was applied to the pictures in Adobe Photoshop.

Spreading assay

Spreading assays were performed as described [7] with modifications. MV^{D7} EGFP and EGFP-Mena isoform expressing cells were trypsinized, re-plated on coverslips coated with laminin (20 μg/ml, Southern Biotech), allowed to spread for 20 minutes, and fixed in cold methanol for 15 minutes at -20°C. Coverslips were washed twice with PBS, cells were permeabilized for 3 minutes at room temperature with 0.2% Triton-X 100 in PBS, washed twice in PBS, and incubated with the mouse monoclonal Fascin antibody to stain filopodia. Cell phenotypes were analyzed and categorized in cells with or without filopodia.

Microscopy

Differential interference contrast (DIC) microscopy

For live cell imaging experiments, cells were plated on glass bottom dishes (MatTek Corporation), treated with 1M HCl for 5 minutes, followed by 70% ethanol and PBS

washes. Cells were imaged with an ORCA-ER camera (Hammamatsu) attached to a Nikon TE300 microscope, using either 10x DIC/0.30NA or 40xDIC/1.3NA Nikon objectives. During time lapse, MTLn3 cells were kept at 37°C with aid of a Solent Incubator chamber (Solent Inc.) fitted for the microscope. All images were collected, measured and compiled with Metamorph imaging software (Molecular Devices) and ImageJ.

Deconvolution wide field fluorescent microscopy

Cells were plated on glass coverslips coated with 100 μg/ml rat-tail Collagen type I (BD bioscience), or 10 μg/ml bovine plasma fibronectin (for MV^{D7} cells) (Sigma), fixed in 4% paraformaldehyde in cytoskeleton buffer (10 mM MES, pH 8.0, 3 mM $MgCl_2$, 138 mM KCl, 2 mM EGTA, pH 6.1, 0.32 M sucrose) for 20 minutes at room temperature, permeabilized in 0.2% Triton X-100 in PBS, blocked in 10% BSA in PBS for 1 hour; incubated with antibodies (indicated in figures and legends) for 1 hour at 37°C, washed 3 times in PBS and incubated with fluorescently labeled secondary antibodies, phalloidin or Hoechst, to visualize F-actin or DNA, respectively. To visualize Mena and Mena11a at the cell-cell junctions (Figure 2A and 2B), cells were permeabilized on ice in cytoskeleton buffer containing 0.2% Triton X-100 for 2 minutes, and fixed on ice in 4% paraformaldehyde in cytoskeleton buffer for 20 minutes.

For tissue staining, 5 μm sections were deparaffinized in xylene, treated with a graded series of alcohol, rehydrated in PBS and subjected to heat-induced antigen retrieval in 10 mM citrate buffer (pH 6.0). Sections were preincubated with 10% normal donkey or goat serum in 0.5% Tween-20 for 2 hours at room temperature, incubated with primary antibodies in 1% donkey or goat serum and 0.5% Tween-20 buffer over night at 4°C, washed 3 times in PBS and incubated in fluorescently labeled secondary antibodies (AlexaFluor, Molecular Probes) for 2 hours at room temperature, and in Hoechst to label the DNA.

z-series of cells and tissues were imaged using a Deltavision microscope using SoftWoRx acquisition software (Applied Precision) or a Nikon Ti inverted microscope

using NIS Elements acquisition software (Nikon), a 40X and 60X 1.4 NA Plan-Apochromat objective lens (Olympus) or a 40X 1.15 NA Plan-Apochromat objective lens (Nikon), and a camera (CoolSNAP HQ; Photometrics or a Zyla4.2 sCMOS; Andor, respectively). Images taken with the Deltavision microscope were deconvolved using Deltavision SoftWoRx software and objective-specific point spread function. Images were combined in Adobe Photoshop for presentation.

3-Dimensional Structural Illumination Microscopy.

Cells were imaged with an OMX-3D Super-resolution microscope (Applied Precision/GE) equipped with 405 nm, 488 nm, 594 nm lasers and 3 Photometrics Cascade II, EMCCD cameras. Images were acquired with a 100X, NA 1.4 oil objective, at 0.125 μm z step, using 1.512 immersion oil. All images were acquired under the same illumination settings (405 nm laser at 19% strength, for 100 msec, 488 nm laser at 1% strength for 150 msec, and 594 nm laser at 50% strength for 100 msec) and then processed with OMX softWoRx software (Applied Precision). Images were saved as .tiff of maximum projections of 8 x 0.125 micron z section stack.

Image quantification

Signal intensities from antibodies or rhodamine-labeled barbed ends along the cell edge were quantified with a published contour-based ImageJ macro [8]. We measured the distribution of signal along the membrane plotted as a function of distance from the cell edge (mean ± SEM) and the sum of the intensities in the first 0.65 μm from the cell edge.

Circularity measures were performed by manually tracing the cells at the free edge of the epithelial monolayer, and by using the circularity plugin built in ImageJ. In this analysis, a circularity value of 1 indicates a perfect circle while, as the value approaches 0, it indicates an elongated cell shape.

For quantitative imaging, we used the same hardware configuration and exposure time for all cell lines analyzed in the same experiment. Manual tracing of cells at the free

edge of the epithelial monolayer was used with the circularity plugin built in ImageJ to derive circularity measures. Extracted pixel intensities were exported to Microsoft Excel for analysis and Graph-Pad Prism for graphing. Figures were assembled in Adobe Illustrator.

Quantitative analysis of fluorescence intensity at cell-cell contacts was performed as described in [9], with modifications. Using the line scan function of ImageJ, a line 4 μm in length (averaged over 20 pixels) was positioned upon randomly chosen contacts. The plot profile feature of ImageJ was used to obtain numerical values for the fluorescence intensity profile along this line; the baseline of each independent profile was corrected by subtracting a constant value from each of the intensity profiles. A minimum of 30 contacts from three individual experiments was measured. The data were imported into Prism 5 and fitted to a Gaussian function with an offset variable. Peak values and their SEs were obtained by nonlinear regression. The full width at half-maximum (FWHM), representing the lateral distribution of E-cadherin, was calculated for each curve (FWHM = standard deviation multiplied by 2.3548). For each experiment, the mean and the SE of the FWHM were calculated.

Immunoprecipitation/tandem mass spectrometry

MTLn3 cells were cultured in 15 cm dishes, starved for 4 hours in L15 media (Gibco) supplemented with 0.35% BSA, stimulated with 5nM EGF, and solubilized immediately after in 350 μl ice cold RIPA buffer (0.1% SDS, 1% NP40, 150 mM NaCl, 50 mM TRIS (pH 8.0), 0.5% Na-deoxycholate, phosphatase inhibitors (PhosStop, Roche), and Complete mini protease inhibitor cocktail tablet (Roche)). Solubilized and insolubilized material were centrifuged at 10,000 rpm for 15 min at 4°C. Supernatants were removed, pre-cleared with Protein A Plus Agarose beads (Pierce), and immunoprecipitated with a rabbit polyclonal antibody to GFP (BD Biosciences) and a rabbit IgG control antibody. The antibody-antigen mixture was mixed with protein A Sepharose beads (Pierce) and washed extensively. Bound protein was solubilized in Laemmli sample buffer and run on 4-15% gradient polyacrylamide gels (BioRad) prior to Coomassie blue staining. Bands were cut out of the gel and sent to the Taplin Biological

Mass Spectrometry Facility (Harvard Medical School) to identify post-translational modifications using HPLC-MS/MS. MS analysis was conducted by the Swanson Biotechnology Proteomics Core (Koch Institute for Integrative Cancer Research, MIT).

Clinical data and analysis

Exon-level gene expression data (RNAseqV2) and clinical data for 1098 breast cancer patients (BRCA) and 461 colorectal adenocarcinoma patients (COAD) were accessed from The Cancer Genome Atlas (TCGA) public data portal (https://tcga-data.nci.nih.gov/tcga/). All clinical samples were de-identified and handled in accordance with the MIT Committee On the Use of Humans as Experimental Subjects (COUHES) Protocol # 0904003185.

MenaCalc was calculated with the following formula:

MenaCalc = average RPKM constitutive exons (hg19 225695653:225695719 and 225688694: 225688772) – RPKM alternate exon 11a (hg19 225692693: 225692755)

The association between MenaCalc, Mena, and Mena11a and metastasis in the COAD cohort was evaluated by logistic regression in R 2.15.3. We first excluded subjects without assignment of pathological stage of metastasis. In order to compare coefficients across tests, we standardized MenaCalc, Mena, and Mena11a RPKM values with mean zero and standard deviation one. Logistic regressions were carried out by choosing the stage of metastasis as the dependent variable (M0 as no evidence of distant metastasis, M1 as evidence with distant metastasis). The only independent variable fitted in the model was MenaCalc, or Mena, or 11a respectively. P values and coefficients corresponding to the independent variables were used to judge the level of association.

For measure of pairwise gene association in the COAD cohort, Spearman's rank correlation coefficients and two-tailed p-values were estimated. The top 50 genes significantly correlating with Mena, Mena11a, and MenaCalc were run through GO

analysis using the Enrichr analysis tool (http://amp.pharm.mssm.edu/Enrichr/) [10] and GSEA using the MsigDb (http://www.broadinstitute.org/gsea/msigdb/index.jsp) [11].

Supplementary Figure Legends

Figure S1

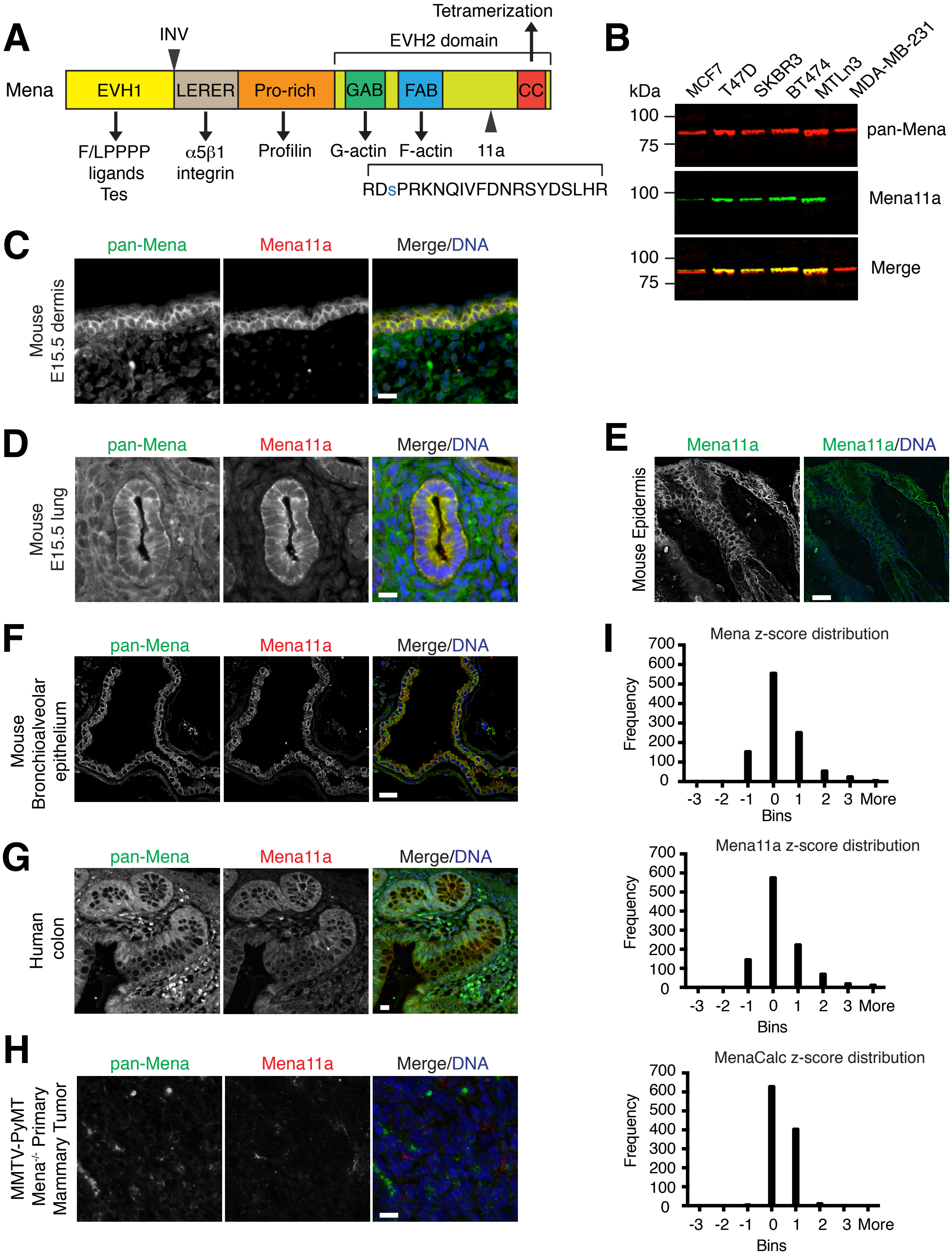

Mena11a expression is restricted to epithelial tissues and epithelial-like cancer cell lines.

A) Mena domains and interacting partners. The EVH1 domain interacts with proteins containing binding sites with a "(F/L) PXϕP" consensus motif (where X is any residue, and ϕ is a hydrophobic residue) and with Tes, a protein without the motif. The proline-rich region has high affinity binding sites for profilin and proteins containing -SH3 and -WW domains; the EVH2 domain contains a G-actin binding site (GAB), F-actin binding site (FAB), and a C-terminal coiled-coil that mediates tetramerization (CC). Mena has several alternatively spliced exons involved in tumor progression; the INV exon is alternatively included next to the "LERER repeat," which directly interacts with α5 integrin; the 11a exon is alternatively included between the FAB and CC. (B) Western blot analysis of endogenous Mena11a and pan-Mena expression in a panel of human breast cancer cell lines (MCF7, T47D, SKBR3, BT474, MDA-MB-231) and in MTLn3 cells. Immunofluorescence of Mena11a and pan-Mena in (C) mouse E15.5 dermis, (D) mouse E15.5 lung epithelium, (E) mouse adult epidermis, (F) mouse adult bronchioalveolar epithelium, (G) adult human colon, (H) primary mammary tumor section from MMTV-PyMT $Mena^{-/-}$ mice. (C)-(H): DNA is visualized with Hoechst staining. Images representative of three independent experiments. Scale bar, 20 μm. (I) z-score distributions for Mena, Mena11a, and MenaCalc values in the BRCA cohort. n = 1060 patients.

Figure S2

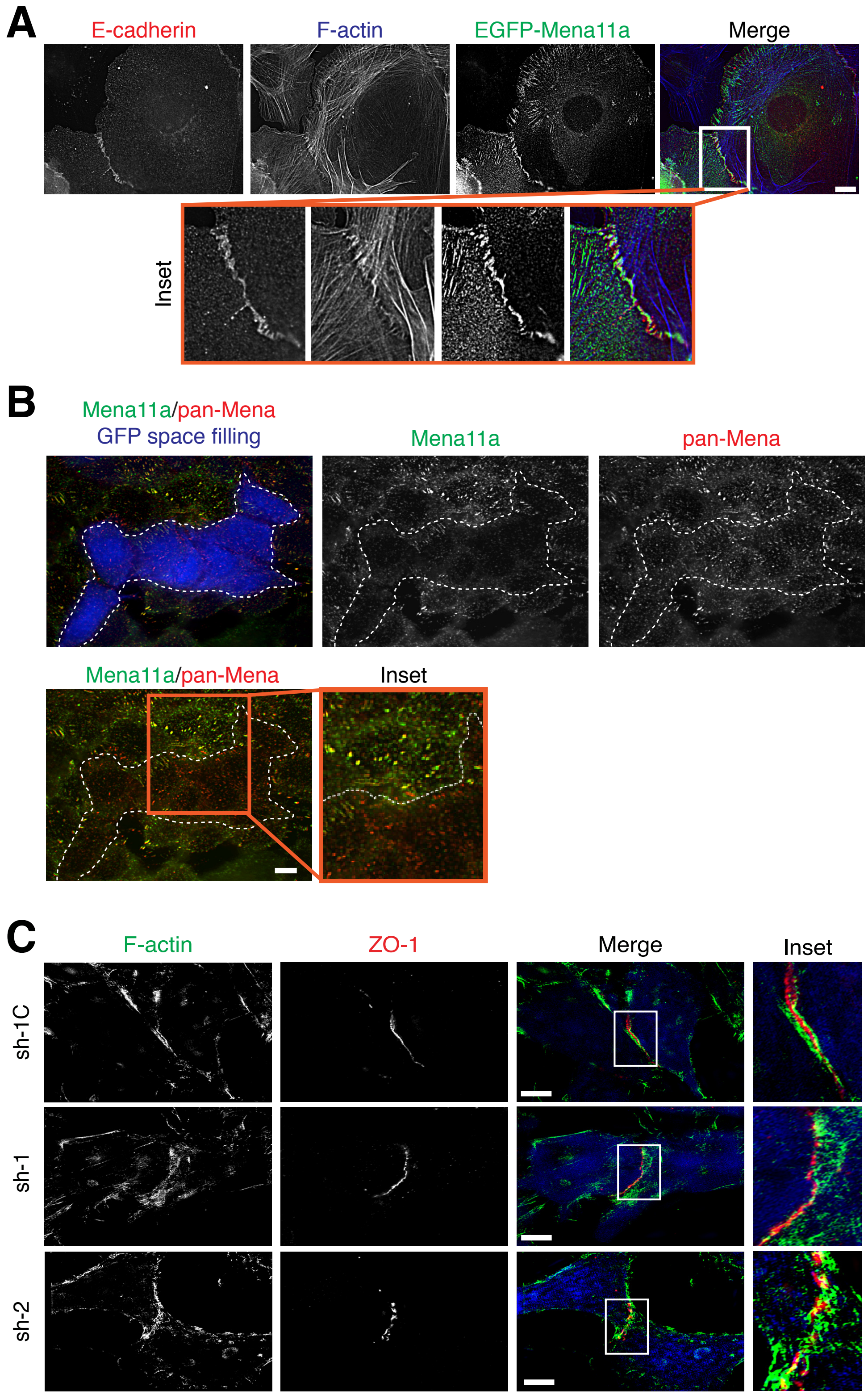
A
E-cadherin
F-actin
EGFP-Mena11a
Merge
Inset
B
Mena11a/pan-Mena
GFP space filling
Mena11a
pan-Mena
Mena11a/pan-Mena
Inset
C
F-actin
ZO-1
Merge
Inset
sh-1C
sh-1
sh-2

Mena11a expression maintains cell-cell junction integrity.

(A) Immunofluorescence of mouse epidermal keratinocytes stably expressing EGFP-Mena11a, immunostained for E-cadherin 3 hours after $CaCl_2$ addition in the culture media, to stimulate junction formation. F-actin visualized by phalloidin labeling. Inset: 7X magnification, demonstrating Mena11a localization to adherens junctions. Scale bar, 10 μm. (B) Immunofluorescence of Mena11a-specific stable knockdown in MCF7 cells using Mena11a and pan-Mena antibodies. Space-filling GFP in blue indicates cells containing the Mena11a knockdown plasmid. Scale bar, 10 μm. Inset: 3X magnification. Mena11a downregulation does not affect Mena protein levels and localization at focal adhesions. (C) 3D-SIM images of ZO-1 in MCF7 cells with isoform-specific knockdown of Mena11a, using two different shRNAs (sh-1, sh-2) and control shRNA (sh-1C). F-actin visualized by phalloidin labeling. Inset: 7X magnification. Scale bar, 10 μm.

Figure S3

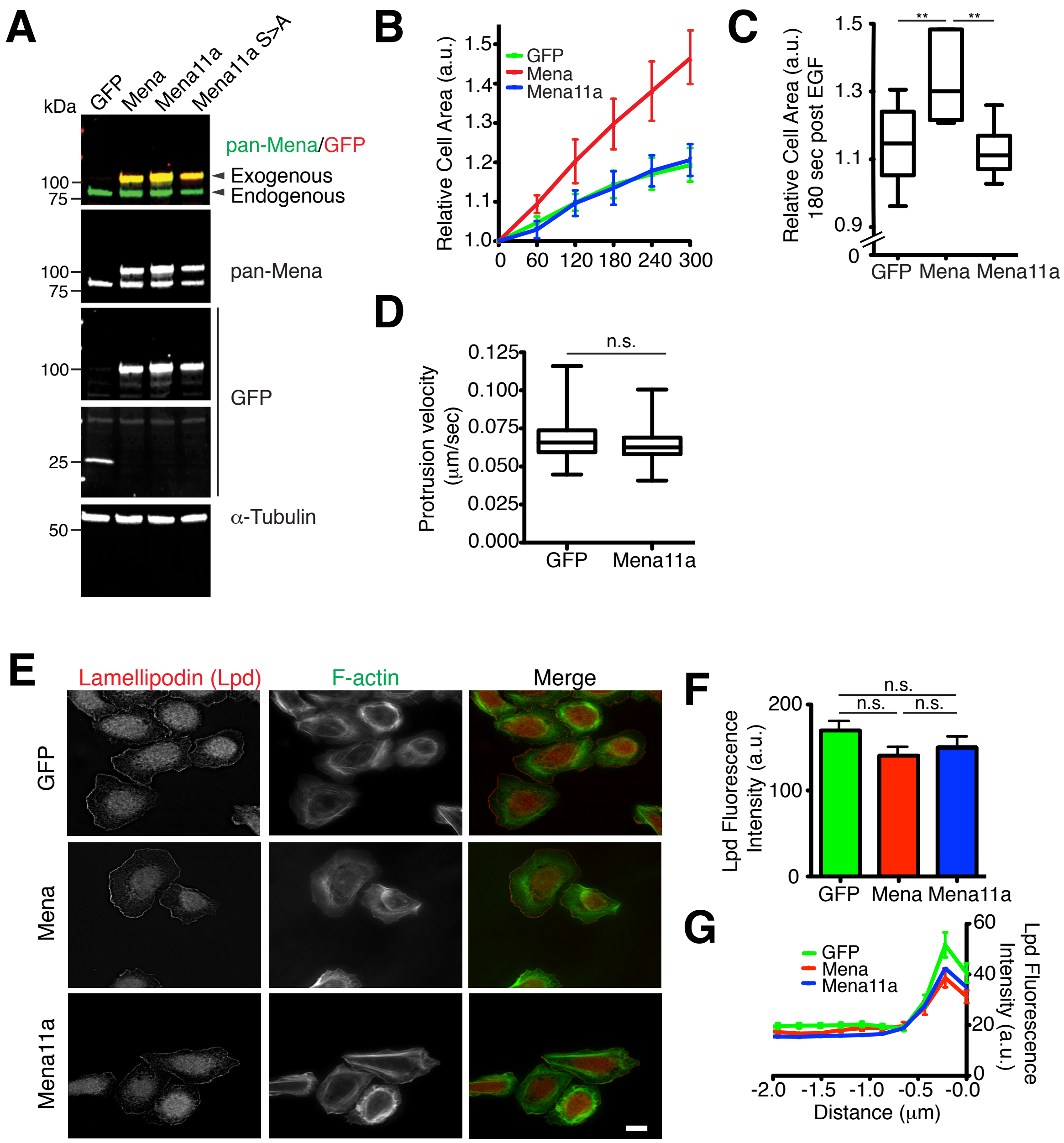
A
GFP
Mena
Mena11a
Mena11a S>A
kDa
pan-Mena/GFP
Exogenous
Endogenous
100
75
pan-Mena
100
75
100
GFP
25
α-Tubulin
50
B
Relative Cell Area (a.u.)
GFP
Mena
Mena11a
1.5
1.4
1.3
1.2
1.1
1.0
0 60 120 180 240 300
C
Relative Cell Area (a.u.) 180 sec post EGF
1.5
1.3
1.1
0.9
0
**
**
GFP Mena Mena11a
D
Protrusion velocity (µm/sec)
n.s.
0.125
0.100
0.075
0.050
0.025
0.000
GFP Mena11a
E
Lamellipodin (Lpd)
F-actin
Merge
GFP
Mena
Mena11a
F
Lpd Fluorescence Intensity (a.u.)
n.s.
n.s.
n.s.
200
100
0
GFP Mena Mena11a
G
GFP
Mena
Mena11a
Lpd Fluorescence Intensity (a.u.)
60
40
20
0
-2.0 -1.5 -1.0 -0.5 -0.0
Distance (µm)

Mena11a expression modulates lamellipodial dynamics but does not affect Lamellipodin recruitment to the leading edge.

(A) Western blot analysis of MTLn3 cells stably expressing GFP (control), Mena, Mena11a, and Mena11a S>A. Membranes were probed with anti pan-Mena and anti GFP antibodies. α-Tubulin is used as loading control. (B) Membrane protrusion kinetics of MTLn3 cells stably expressing GFP, Mena, or Mena11a following 0.5nM EGF stimulation. Error bars represent SEM. (C) Membrane protrusion of MTLn3 cells stably expressing GFP, Mena, and Mena11a after 0.5nM EGF stimulation at t = 180 seconds. Center-line of box indicates the median, top indicates 75th quartile, bottom indicates 25th quartile. Whiskers represent 90th and 10th percentiles. Error bars represent SEM. Results from triplicates, >90 cells analyzed. One-way ANOVA $^{**}p<0.01$.

(D) Box and whisker plot of velocity of individual protrusion events for MTLn3 cells stably expressing GFP and Mena11a during stimulation with 5nM EGF. Center-line of box indicates median, top indicates 75th quartile, bottom indicates 25th quartile. Whiskers represent 10th and 90th percentiles. Data for MTLn3-GFP cells come from 112 events of protrusion; for MTLn3 EGFP-Mena11a, 90 events of protrusion. n.s.: non significant. (E) Immunofluorescence of endogenous Lamellipodin (Lpd) in MTLn3 cells stably expressing GFP, Mena11a after stimulation with 5nM EGF for 180 seconds. F-actin visualized by phalloidin labeling. Scale bar, 10 μm. (F) Quantification of Lamellipodin (Lpd) fluorescence intensity sum of the initial 0.65 μm from the leading edge of MTLn3 cells stably expressing GFP, Mena and Mena11a. a.u. = arbitrary units. Error bars: SEM. Results represent triplicates, >30 cells analyzed. One-way ANOVA, n.s.: not significant. (G) Normalized pixel intensities of Lpd plotted as a function of

distance from the cell edge (mean ± SEM). Results represent triplicates, over 30 cells analyzed. Panels G-I show that Mena11a expression does not significantly affect Lpd recruitment to the leading edge of protruding lamellipodia.

Figure S4

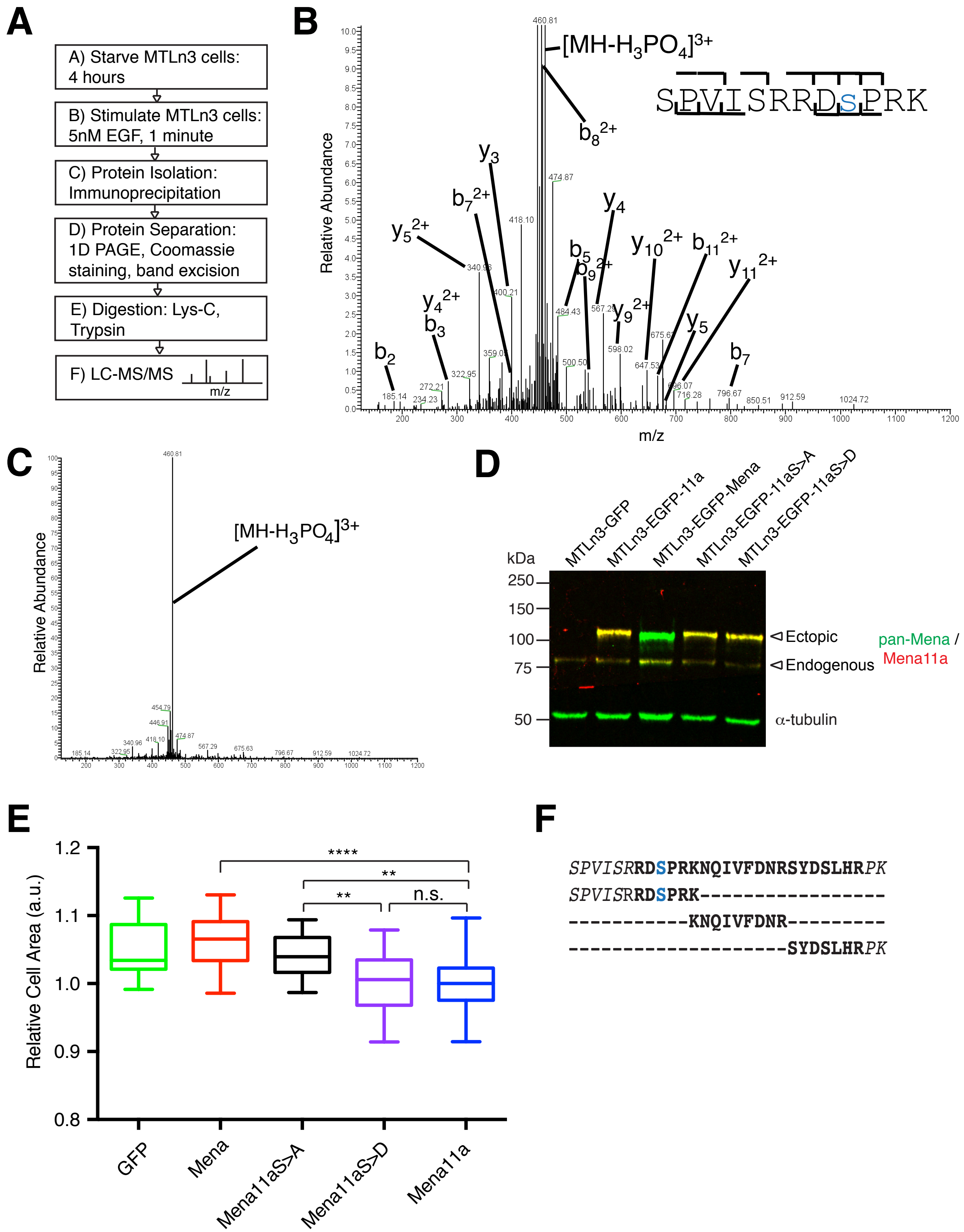
A
A) Starve MTLn3 cells: 4 hours
B) Stimulate MTLn3 cells: 5nM EGF, 1 minute
C) Protein Isolation: Immunoprecipitation
D) Protein Separation: 1D PAGE, Coomassie staining, band excision
E) Digestion: Lys-C, Trypsin
F) LC-MS/MS
m/z
B
[MH-H3PO4]3+
SPVISRRDsPRK
Relative Abundance
m/z
C
[MH-H3PO4]3+
Relative Abundance
D
MTLn3-GFP
MTLn3-EGFP-11a
MTLn3-EGFP-Mena
MTLn3-EGFP-11aS>A
MTLn3-EGFP-11aS>D
kDa
250
150
100
75
50
Ectopic
Endogenous
α-tubulin
pan-Mena / Mena11a
E
Relative Cell Area (a.u.)
****
**
**
n.s.
GFP
Mena
Mena11aS>A
Mena11aS>D
Mena11a
F
SPVISRRDSPRKNQIVFDNRSYDSLHRPK
SPVISRRDSPRK-----------------
-----------KNQIVFDNR---------
--------------------SYDSLHRPK

Characterization of the Mena11a serine phosphorylation site.

(A) Strategy for immunoprecipitation (IP)/tandem-mass spectrometry (MS/MS) of EGFP-Mena11a from MTLn3 cell lysates after 5nM EGF stimulation for 60 seconds.

(B) MS/MS spectrum of phospho-peptide SPVISRRDsPRK (zoomed in for peak detail). Ions labeled with -H_3PO_4 indicate a neutral loss of phosphoric acid. “b” and “y” ion series represent fragment ions containing the N- and C-termini of the peptide, respectively. (C) MS/MS spectrum of phospho-peptide. Ion labeled with -H_3PO_4 indicates a neutral loss of phosphoric acid. (D) Western blot analysis of MTLn3 cells stably expressing GFP (control), Mena, Mena11a, Mena11a S>A, and Mena11a S>D. Membranes were probed with anti Mena11a and anti pan-Mena antibodies. α-tubulin was used as a loading control. Upper arrowhead = ectopic expression of GFP-tagged protein. Lower arrowhead = endogenous expression of protein. (E) Membrane protrusion of MTLn3 cells stably expressing GFP, Mena, Mena11a, Mena11a S>A, and Mena11a S>D after stimulation with 5 nM EGF for 60 seconds. Center-line of box indicates median, top indicates 75th quartile, bottom indicates 25th quartile. Whiskers represent 10th and 90th percentiles. Error bars: SEM. Results represent five experiments, >20 cells analyzed for each condition. One-way ANOVA **$p<0.01$, ****$p<0.001$, n.s. not significant. (F) Peptides from (IP)/tandem-mass spectrometry (MS/MS) of EGFP-Mena11a from MTLn3 cell lysates after 5nM EGF stimulation for 60 seconds demonstrating coverage of Mena11a 21 amino acid insertion. Serine in blue is phosphorylated.

Supplementary Movie Legends

Supplementary Movie S1

Membrane Protrusion of EGF-stimulated MTLn3-GFP, GFP-Mena11a and GFP-Mena cells.

MTLn3 cells stably expressing GFP, GFP-Mena11a, and GFP-Mena were stimulated with 5nM EGF for 300 seconds (starting at 60 seconds). Images were analyzed by time-lapse differential interference contrast microscopy (TE300, Nikon). Frames were taken every 3 seconds for 360 seconds.

Supplementary Movie S2

Membrane Protrusion of EGF-stimulated MTLn3 GFP-Mena11a and GFP-Mena11aS>A cells.

MTLn3 cells stably expressing GFP-Mena11a and GFP-Mena11aS>A were stimulated with 5nM EGF for 300 seconds (starting at 60 seconds). Images were analyzed by time-lapse differential interference contrast microscopy (TE300, Nikon). Frames were taken every 3 seconds for 360 seconds.

Supplementary Table S1: Top 150 genes correlating with ENAH (Mena), Mena11a, and MenaCalc in COAD cohort

ENAH (Mena)			Mena11a			MenaCalc		
Gene Symbol	Spearman Correlation	p-value	Gene Symbol	Spearman Correlation	p-value	Gene Symbol	Spearman Correlation	p-value
ENAH	0.887	2.4126E-162	ENAH	0.763	1.29734E-92	CALD1	0.664	2.37083E-62
ZNF281	0.581	1.08034E-44	C1orf107	0.539	1.38584E-37	PKD2	0.654	7.39677E-60
DSTYK	0.569	1.32555E-42	HEATR1	0.515	6.11056E-34	ZNF281	0.647	2.29211E-58
ASH1L	0.568	2.88934E-42	SMARCC1	0.502	5.61988E-32	FILIP1L	0.646	4.20101E-58
CAMSAP1L1	0.562	2.56955E-41	POGK	0.493	1.06818E-30	CSGALNACT2	0.644	1.10649E-57
TEAD1	0.556	2.26909E-40	PIGM	0.485	1.01278E-29	ZEB1	0.643	3.03204E-57
TRIM44	0.556	2.68055E-40	PRKDC	0.476	1.50279E-28	MPDZ	0.634	2.77011E-55
ARID4B	0.554	6.72877E-40	DHX9	0.473	4.16224E-28	LPHN2	0.632	8.04148E-55
TAOK1	0.553	7.20728E-40	RBBP5	0.470	8.46346E-28	FERMT2	0.629	2.70265E-54
RIF1	0.552	1.25974E-39	NUP133	0.469	1.17372E-27	TCF4	0.627	6.72683E-54
RBBP5	0.551	1.90426E-39	ZMYM4	0.467	1.98935E-27	MSRB3	0.626	1.69329E-53
ZMYM4	0.549	3.48196E-39	NF1	0.467	2.14235E-27	MLLT11	0.621	1.37394E-52
POGK	0.543	3.92292E-38	TOP2B	0.461	1.31048E-26	AKT3	0.619	3.60755E-52
UBXN7	0.542	6.3511E-38	ASH1L	0.460	1.77648E-26	SGCD	0.618	6.91996E-52
ASXL2	0.538	2.46811E-37	FNBP1L	0.460	1.85283E-26	QKI	0.618	8.52829E-52
RAB3GAP2	0.537	2.99968E-37	RAB3GAP2	0.459	2.37054E-26	DDR2	0.617	1.31648E-51
SP3	0.537	3.30897E-37	VPS54	0.459	2.38285E-26	FSTL1	0.614	4.65926E-51
LAMC1	0.537	3.75118E-37	DARS2	0.457	3.4254E-26	RAI14	0.613	9.13952E-51
ATRX	0.535	7.66614E-37	BRWD3	0.456	5.00787E-26	CEP170	0.611	1.85436E-50
HEATR1	0.534	9.49691E-37	MED1	0.452	1.37538E-25	ZNF521	0.610	2.52141E-50
MED1	0.533	1.49656E-36	NLN	0.451	1.87979E-25	ZEB2	0.609	5.09707E-50
CEP350	0.531	3.20474E-36	ZNF146	0.450	2.52895E-25	KIAA1462	0.609	5.19609E-50
NF1	0.530	4.06675E-36	TEX2	0.450	2.75029E-25	SYT11	0.608	6.52358E-50

MAP3K2	0.519	1.59368E-34	STT3B	0.448	5.14748E-25	FRMD6	0.607	1.4236E-49
MAN1A2	0.518	2.25312E-34	IPO7	0.445	1.09733E-24	DZIP1	0.604	3.77822E-49
HIP1	0.516	5.71967E-34	MEX3A	0.445	1.1382E-24	FAM171B	0.604	4.58377E-49
PIK3C2A	0.516	5.99203E-34	RIF1	0.444	1.40932E-24	AKAP12	0.603	5.94818E-49
C1orf107	0.516	6.01261E-34	HOOK1	0.442	2.46833E-24	ELK3	0.603	7.84746E-49
CLASP2	0.515	8.36349E-34	TRIM44	0.439	4.36833E-24	NEXN	0.601	1.96656E-48
EPC2	0.514	1.01256E-33	RBM12	0.439	4.79628E-24	STON1	0.601	2.02797E-48
MYO9A	0.512	1.70974E-33	LASS6	0.439	5.28588E-24	ANTXR1	0.598	8.11901E-48
ZBTB41	0.511	2.74431E-33	CDK12	0.439	5.40613E-24	ECM2	0.597	1.17495E-47
ATF6	0.511	3.24201E-33	WDR3	0.438	5.65046E-24	FGF7	0.597	1.23325E-47
MTR	0.510	3.80632E-33	FLVCR1	0.438	7.17792E-24	FBN1	0.596	1.51373E-47
CREB1	0.508	8.1241E-33	ZNF704	0.437	7.91286E-24	VGLL3	0.596	1.71094E-47
TOP2B	0.507	9.47136E-33	QTRTD1	0.436	1.09421E-23	COL8A1	0.595	2.8158E-47
SBF2	0.505	1.84023E-32	SBF2	0.433	2.20468E-23	CACNA2D1	0.595	2.94103E-47
MED13L	0.504	2.64888E-32	RALGAPA2	0.433	2.23694E-23	ZNF532	0.594	3.42341E-47
RAB11FIP2	0.501	6.24528E-32	GPATCH2	0.432	2.96439E-23	MAP1B	0.594	4.01212E-47
CDK12	0.500	1.17287E-31	ZBTB41	0.431	3.54958E-23	LRCH2	0.592	9.95183E-47
PTPN11	0.499	1.305E-31	CLASP2	0.430	5.07515E-23	OSMR	0.592	1.03851E-46
KLHDC10	0.499	1.31174E-31	SP3	0.428	8.69286E-23	TRPS1	0.592	1.10863E-46
BAT2L2	0.497	2.23087E-31	MBTD1	0.427	1.08966E-22	ADAMTS12	0.592	1.14467E-46
WNK1	0.497	2.26094E-31	SMC6	0.427	1.18642E-22	CLIC4	0.592	1.15459E-46
UBR3	0.497	2.56563E-31	SYDE2	0.426	1.38032E-22	GUCY1A3	0.591	1.28504E-46
ABI2	0.497	2.95602E-31	SRPK2	0.426	1.4943E-22	FKBP14	0.590	2.80408E-46
DHX36	0.494	6.15222E-31	ATRX	0.425	1.76976E-22	ELOVL4	0.589	3.09138E-46
NCOA3	0.494	6.2791E-31	SUZ12	0.425	1.84542E-22	GPR116	0.589	3.16064E-46
STRN	0.493	8.76582E-31	UBR3	0.425	1.92999E-22	MCC	0.589	4.01398E-46
RPS6KC1	0.493	9.45052E-31	GSK3B	0.423	2.95559E-22	ASAM	0.589	4.2853E-46
RPRD2	0.492	1.3453E-30	TMEM192	0.422	3.43055E-22	VCAN	0.588	6.04336E-46

PBRM1	0.491	1.59826E-30	ZNF445	0.422	3.98128E-22	PHLDB2	0.588	6.7414E-46
ANKRD50	0.491	1.60134E-30	WDR35	0.422	4.03154E-22	COL5A2	0.587	8.22525E-46
ZNF192	0.491	1.6557E-30	KLHDC10	0.422	4.25633E-22	LAMC1	0.587	1.08222E-45
BRAF	0.491	1.71574E-30	TRIM24	0.421	4.35663E-22	SDC2	0.586	1.38174E-45
MGA	0.490	2.23182E-30	UTP20	0.420	5.58506E-22	FILIP1	0.586	1.54122E-45
AKAP2	0.489	2.75401E-30	BAT2L2	0.420	6.67861E-22	FLRT2	0.585	2.14193E-45
DIXDC1	0.489	3.10762E-30	AHCTF1	0.420	6.76719E-22	GUCY1B3	0.584	3.42301E-45
SLC4A7	0.489	3.65789E-30	BRAF	0.419	7.05786E-22	RGL1	0.584	3.65538E-45
VPS54	0.488	3.8834E-30	STAU2	0.419	8.05224E-22	RASSF8	0.583	4.39932E-45
TBCEL	0.488	3.9216E-30	KIAA1147	0.419	8.36421E-22	CALCRL	0.583	4.83548E-45
BMPR2	0.488	4.40756E-30	ROCK2	0.418	9.18526E-22	RAB31	0.583	4.90615E-45
MED13	0.487	5.46157E-30	PREPL	0.418	1.05355E-21	CYP7B1	0.581	1.01952E-44
TP53BP2	0.487	5.85287E-30	DDX21	0.418	1.06812E-21	MYCT1	0.580	1.52488E-44
ZAK	0.487	6.77671E-30	STRN	0.417	1.14595E-21	GEFT	0.580	1.54275E-44
C5orf42	0.486	8.03555E-30	RALGAPB	0.417	1.30947E-21	MYLK	0.579	2.3047E-44
KIAA0947	0.486	8.87187E-30	RBM12B	0.416	1.45912E-21	GNB4	0.579	2.32251E-44
NUP133	0.485	1.07258E-29	RRP15	0.416	1.47842E-21	RNF150	0.579	2.73868E-44
PIK3CA	0.485	1.13924E-29	DHX36	0.416	1.51267E-21	EFHA2	0.578	3.41784E-44
DHX9	0.485	1.16443E-29	ANKRD17	0.416	1.65512E-21	COL15A1	0.577	5.16811E-44
BRWD3	0.485	1.16904E-29	ADNP	0.416	1.67843E-21	SULF1	0.577	5.27847E-44
TRIM33	0.483	1.79691E-29	KLHL23	0.416	1.78335E-21	C20orf194	0.577	5.33527E-44
KIDINS220	0.483	1.79706E-29	C14orf106	0.415	2.01301E-21	LAMA4	0.577	5.9781E-44
PIK3R4	0.483	2.27871E-29	ZNF664	0.415	2.15173E-21	SPARCL1	0.577	6.54057E-44
TRIP12	0.482	2.43368E-29	COBLL1	0.414	2.6328E-21	DLC1	0.576	8.5575E-44
ANKRD36BP1	0.482	2.72493E-29	LRRC58	0.413	3.03087E-21	SPG20	0.575	1.44213E-43
TOR1AIP1	0.482	2.86992E-29	SMARCA5	0.413	3.38268E-21	JAZF1	0.575	1.46716E-43
KLHL20	0.481	3.42775E-29	SR140	0.411	5.52414E-21	HEG1	0.575	1.59902E-43
CNST	0.481	3.9317E-29	ZKSCAN1	0.411	5.56016E-21	COL12A1	0.574	2.06174E-43

SMARCC1	0.480	4.70882E-29	MRE11A	0.411	5.82705E-21	BNC2	0.573	2.57761E-43
ZNF146	0.480	4.74834E-29	CHD7	0.411	5.88975E-21	LOC399959	0.573	3.21368E-43
MOBKL1A	0.480	5.58631E-29	CEP350	0.410	6.58421E-21	IL6ST	0.573	3.28444E-43
RALGAPB	0.480	5.63176E-29	RPRD2	0.408	1.23364E-20	PLN	0.572	4.59466E-43
WDR35	0.479	6.24651E-29	PTPN11	0.407	1.32048E-20	GJC1	0.571	6.44272E-43
GPATCH2	0.478	9.09821E-29	PRPF40A	0.407	1.39716E-20	LCA5	0.571	7.88276E-43
SRPK2	0.478	9.54852E-29	GPR89A	0.407	1.40186E-20	GPC6	0.570	8.71399E-43
REST	0.478	9.59308E-29	SMEK2	0.407	1.58285E-20	ZFHX4	0.570	9.34884E-43
PARD3B	0.477	1.10394E-28	CBX1	0.406	1.79486E-20	ITPR1	0.570	9.79887E-43
PPP4R2	0.477	1.285E-28	NAALADL2	0.406	1.85124E-20	ELTD1	0.569	1.65252E-42
ASAP1	0.476	1.54466E-28	PIK3C2A	0.406	1.86117E-20	MRVI1	0.569	1.88362E-42
PIKFYVE	0.476	1.57714E-28	ARFGEF1	0.406	1.89145E-20	AMOTL1	0.568	2.36213E-42
ABL2	0.476	1.81077E-28	TRIM33	0.406	1.95006E-20	IL1R1	0.568	2.74751E-42
GIGYF2	0.475	1.96148E-28	UBR5	0.405	2.08629E-20	CCDC88A	0.567	2.99764E-42
VASH2	0.475	2.16407E-28	ASXL2	0.405	2.20385E-20	RNF180	0.567	3.95189E-42
BPTF	0.475	2.42271E-28	XPR1	0.405	2.33493E-20	SGIP1	0.567	4.02056E-42
SOS1	0.475	2.45416E-28	SAMD12	0.405	2.52688E-20	KLHL5	0.567	4.08719E-42
XPR1	0.475	2.46869E-28	POLR1B	0.404	2.56326E-20	TSPYL5	0.566	4.70399E-42
UHMK1	0.473	3.44825E-28	CEP68	0.404	2.90336E-20	TMEM47	0.565	6.77036E-42
SMARCA5	0.473	3.56799E-28	HNRNPU	0.404	3.18937E-20	GREM1	0.565	8.04899E-42
KCTD20	0.473	3.77855E-28	URB2	0.403	3.24508E-20	MITF	0.564	9.83851E-42
PIGM	0.473	4.28039E-28	PPP4R2	0.403	3.67936E-20	NAP1L3	0.564	1.03779E-41
GSK3B	0.472	4.93214E-28	TAOK1	0.403	3.80782E-20	ASPN	0.564	1.04674E-41
ZNF148	0.471	6.66088E-28	ARID4B	0.403	3.86538E-20	NRP1	0.564	1.18332E-41
ZKSCAN1	0.471	6.72386E-28	NEU3	0.403	3.97477E-20	ITGB1	0.564	1.32714E-41
CHD6	0.470	1.00758E-27	KIAA1804	0.402	4.84874E-20	TNS1	0.563	1.45833E-41
ZFP106	0.470	1.09195E-27	CCDC47	0.401	5.13875E-20	PALMD	0.563	1.88946E-41
CDKL5	0.469	1.27063E-27	SOCS7	0.401	5.20927E-20	FAM126A	0.562	2.43006E-41

CHD9	0.469	1.3135E-27	CEBPZ	0.401	5.31519E-20	CAMSAP1L1	0.562	2.91253E-41
PPPDE1	0.469	1.36713E-27	TTC37	0.401	5.42737E-20	SMARCA1	0.562	3.0325E-41
MKL2	0.469	1.36739E-27	ZZZ3	0.401	5.71517E-20	FAM129A	0.561	3.19659E-41
WDFY3	0.469	1.42725E-27	DDX18	0.401	5.8829E-20	MAP1A	0.561	3.545E-41
IL6ST	0.469	1.46017E-27	FKTN	0.401	6.28209E-20	LUM	0.561	3.57319E-41
SDCCAG1	0.468	1.62164E-27	ZNF643	0.400	7.56015E-20	HSPB8	0.561	4.23084E-41
ZYG11B	0.468	1.63172E-27	GIGYF2	0.400	8.03943E-20	NHSL2	0.560	4.94828E-41
SHPRH	0.467	2.32909E-27	CHML	0.399	8.4647E-20	ZFPM2	0.560	5.61816E-41
ATF2	0.467	2.41455E-27	LBR	0.399	8.93354E-20	MMP16	0.560	6.82314E-41
TTBK2	0.466	2.62031E-27	DSTYK	0.399	9.13378E-20	PPP1R3C	0.559	7.12472E-41
USP34	0.466	3.25072E-27	YTHDC1	0.399	9.23443E-20	CFH	0.559	7.22671E-41
HIPK3	0.465	3.86768E-27	GEMIN5	0.398	1.07501E-19	C14orf132	0.558	1.11985E-40
PCNX	0.465	4.28391E-27	OPA1	0.398	1.07682E-19	PABPC4L	0.558	1.13581E-40
TMEM170B	0.464	5.18851E-27	CAPN7	0.398	1.08294E-19	CDK14	0.558	1.3307E-40
HELZ	0.464	5.54316E-27	CELF2	0.398	1.19314E-19	DIXDC1	0.557	1.69855E-40
CCDC75	0.464	5.95382E-27	ZC3H8	0.398	1.26334E-19	NECAB1	0.557	1.72448E-40
KIAA0754	0.464	5.96835E-27	MED13	0.397	1.34668E-19	ENTPD1	0.557	2.06559E-40
UBR5	0.464	6.14206E-27	LMBR1	0.397	1.38573E-19	ADAMTS5	0.556	2.53553E-40
DYNC2H1	0.463	6.64622E-27	NUP155	0.397	1.54204E-19	WWTR1	0.556	2.7124E-40
EXOC6B	0.463	7.14089E-27	ZBTB33	0.397	1.60348E-19	COL6A3	0.556	2.93374E-40
ARNT	0.462	9.58291E-27	ZNF678	0.396	1.6938E-19	SHE	0.556	3.02145E-40
FAM20B	0.461	1.12536E-26	DDX52	0.396	1.73018E-19	PRKD1	0.556	3.07556E-40
RBM12B	0.461	1.12673E-26	INADL	0.396	1.79471E-19	HIP1	0.556	3.08734E-40
NPAT	0.461	1.1619E-26	LRPPRC	0.396	2.00827E-19	EVC	0.555	3.26699E-40
LATS1	0.461	1.19738E-26	ATR	0.395	2.07095E-19	PABPC5	0.555	3.68135E-40
ROCK2	0.461	1.29205E-26	USP46	0.395	2.21948E-19	SPOCK1	0.555	3.68709E-40
ZNF678	0.460	1.77977E-26	APOOL	0.394	2.59158E-19	HMCN1	0.554	4.906E-40
VPS13B	0.459	1.93518E-26	CLCN3	0.394	2.74712E-19	DCLK1	0.554	5.54611E-40

LMBRD2	0.459	2.13783E-26	MOBKL1A	0.393	3.3359E-19	TEK	0.553	7.53317E-40
ANKRD17	0.459	2.43719E-26	ZAK	0.393	3.52799E-19	ITGA1	0.553	7.56485E-40
SP4	0.458	3.2114E-26	TXLNG	0.393	3.66948E-19	GLI3	0.552	1.03536E-39
ERCC4	0.458	3.25485E-26	PPPDE1	0.393	3.75891E-19	FAM26E	0.552	1.07739E-39
NIPBL	0.457	3.41131E-26	PHF20	0.392	4.40852E-19	MEIS1	0.552	1.21676E-39
PHF20	0.457	3.5299E-26	LRRC8D	0.392	4.80326E-19	SNAI2	0.552	1.26255E-39
NUP153	0.457	3.71987E-26	UGGT1	0.392	4.92873E-19	PDGFC	0.551	1.82048E-39
SOCS5	0.457	4.04833E-26	CCDC121	0.391	5.79828E-19	JAM3	0.551	2.01796E-39
RBM12	0.456	4.46128E-26	MTR	0.390	6.26859E-19	TSHZ3	0.550	2.37561E-39
AHCTF1	0.456	4.61288E-26	ZNF619	0.390	7.10179E-19	BEND6	0.550	2.75097E-39
TGFB2	0.456	4.86893E-26	NUFIP2	0.390	7.34929E-19	INHBA	0.550	2.89393E-39
TTPAL	0.456	4.9553E-26	NSF	0.389	8.29015E-19	PAPPA	0.549	3.46807E-39
TAF2	0.456	5.5372E-26	MAN1A2	0.389	8.56492E-19	GJA1	0.549	3.74079E-39
DAAM1	0.456	5.62519E-26	TTPAL	0.389	8.92092E-19	TGFB2	0.549	4.28649E-39
CBX1	0.455	6.0724E-26	ZNF124	0.389	9.057E-19	VEGFC	0.548	5.8342E-39

Supplementary References

1. Bear, J. E. *et al.* Negative regulation of fibroblast motility by Ena/VASP proteins. *Cell* **101,** 717–728 (2000).

2. Philippar, U. *et al.* A Mena invasion isoform potentiates EGF-induced carcinoma cell invasion and metastasis. *Dev. Cell* **15,** 813–828 (2008).

3. Pino, M. S. *et al.* Human Mena+11a isoform serves as a marker of epithelial phenotype and sensitivity to epidermal growth factor receptor inhibition in human pancreatic cancer cell lines. *Clin. Cancer Res.* **14,** 4943–50 (2008).

4. Lanier, L. M. *et al.* Mena is required for neurulation and commissure formation. *Neuron* **22,** 313–325 (1999).

5. Krause, M. *et al.* Lamellipodin, an Ena/VASP ligand, is implicated in the regulation of lamellipodial dynamics. *Dev. Cell* **7,** 571–583 (2004).

6. Svitkina, T. M., Verkhovsky, A. B. & Borisy, G. G. Improved procedures for electron microscopic visualization of the cytoskeleton of cultured cells. *J. Struct. Biol.* 290–303 (1995).

7. Applewhite, D. A. *et al.* Ena/VASP Proteins Have an Anti-Capping Independent

Function in Filopodia Formation. *Mol. Biol. Cell* **18,** 2579–2591 (2007).

8. Cai, L., Marshall, T. W., Uetrecht, A. C., Schafer, D. A. & Bear, J. E. Coronin 1B Coordinates Arp2/3 Complex and Cofilin Activities at the Leading Edge. *Cell* **128,** 915–929 (2007).

9. Leerberg, J. M. *et al.* Tension-Sensitive Actin Assembly Supports Contractility at the Epithelial Zonula Adherens. *Curr. Biol.* 1–11 (2014). doi:10.1016/j.cub.2014.06.028

10. Chen, E. Y. *et al.* Enrichr: interactive and collaborative HTML5 gene list enrichment analysis tool. *BMC Bioinformatics* **14,** 128 (2013).

11. Subramanian, A. *et al.* Gene set enrichment analysis: A knowledge-based approach for interpreting genome-wide expression profiles. *Proc. Natl. Acad. Sci.* **102 ,** 15545–15550 (2005).
